# Expressed disapproval does not sustain long-term cooperation as effectively as costly punishment

**DOI:** 10.1017/ehs.2024.41

**Published:** 2024-12-26

**Authors:** Adam Sparks, Tyler Burleigh, Pat Barclay

**Affiliations:** 1 Department of Psychology, University of Guelph, Guelph, ON, Canada; 2 Independent Researcher, Toronto, ON, Canada

**Keywords:** cooperation, punishment, sanctions, habituation, learning

## Abstract

Punishment plays a role in human cooperation, but it is costly. Prior research shows that people are more cooperative when they expect to receive negative feedback for non-cooperation, even in the absence of costly punishment, which would have interesting implications for theory and applications. However, based on theories of habituation and cue-based learning, we propose that people will learn to ignore expressions of disapproval that are not clearly associated with material costs or benefits. To test this hypothesis, we conducted a between-subjects, 40-round public goods game (i.e. much longer than most studies), where participants could respond to others’ contributions by sending numerical disapproval messages, paying to reduce others’ earnings, or neither. Consistent with previous results, we observed steadily increasing contributions in the costly punishment condition. In contrast, contributions declined after the early rounds in the expressed disapproval condition, and were eventually no higher than the basic control condition with neither costly punishment nor disapproval ratings. In other words, costless disapproval may temporarily increase cooperation, but the effects fade. We discuss the theoretical and applied implications of our findings, including the unexpectedly high levels of cooperation in a second control condition.

## Graphical abstract

**Figure:**
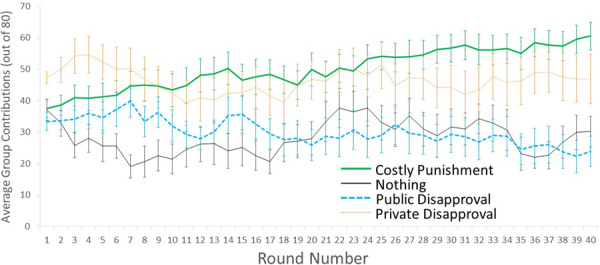

**Social media summary:** Disapproval by itself is not enough to maintain long-term cooperation – punishment must have tangible consequences

## Introduction

People are more cooperative when they can be punished for non-cooperation (see meta-analysis by Balliet et al., 2011). The logic is as follows: the presence of punishment changes people`s incentive structures, such that it pays better to cooperate than to defect and get punished. Given these incentives, multiple authors have argued that punishment was a major factor in the biological evolution of cooperative sentiment, and in the cultural evolution of cooperative institutions (e.g. Boyd et al., 2003; de Quervain et al., 2004; Fehr & Gächter, 2002; Gächter et al., 2008; Gürerk et al., 2006; Henrich et al., 2006; for reviews, see Barclay, in press; Raihani & Bshary, 2019). However, punishment is costly to multiple parties (e.g. Bochet et al., 2006; Noussair & Tucker, 2005). Punishers incur time and energetic costs, a risk of injury and retaliation, and possible loss of relationships with those whom they punish or with observers. Recipients of punishment experience the physical and social harms of being punished. As a result of these costs, punishment reduces group efficiency in the short term (e.g. Bochet et al., 2006; Cinyabuguma et al., 2006) and many interactions are required before these costs are recouped (Gächter et al., 2008). This then leads to optimization questions. How can we use punishment as efficiently as possible? How do we minimize punishment costs while maintaining cooperation? Can costless and/or non-destructive types of punishment be effective?

### Disapproval vs. costly punishment

Some researchers report that merely expressing disapproval can be more effective than costly punishment, or can be similarly effective but cheaper for all parties (e.g. Bochet et al., 2006; Masclet et al., 2003, 2013; Peeters & Vorsatz, 2013). Under this view, people seek to avoid others’ disapproval (‘disapproval aversion’, e.g. Akerlof, 1980; Holländer, 1990; López-Pérez & Vorsatz, 2010; Masclet et al., 2003; Xiao & Houser, 2009), which broadly encompasses a wide variety of negative communication, including informal punishment, threat, moral condemnation/outrage, verbal feedback, non-monetary sanctions, expression of negative emotion, and so on. These experiments typically find that people cooperate more in experimental games where others can provide verbal or non-verbal feedback (which is typically negative towards non-cooperators) than in similar games without such feedback (e.g. Brook & Servátka, 2016; Ellingsen & Johannesson, 2008; Festré & Garrouste, 2014; López-Pérez & Vorsatz, 2010; Masclet et al., 2003; Xiao & Houser, 2009). Under this perspective, disapproval-avoidance alone is sufficient to motivate people to cooperate, and disapproval is generally seen as an isolated phenomenon which is a strict alternative to punishment (e.g. Brook & Servátka, 2016; Fehr & Falk, 2002; Festré & Garrouste, 2014; López-Pérez & Vorsatz, 2010; Masclet et al., 2003, 2013; Xiao & Houser, 2009). More broadly, this body of evidence is often interpreted as fitting with a larger phenomenon whereby communication enhances cooperation.

In contrast, we argue here that punishment will only be effective at maintaining cooperation in the long term if that punishment has tangible consequences – the disapproval has to have ‘teeth’. By itself, disapproval alone has no impact on an offender's fitness. An offender could freely ignore any disapproval that carried no other consequences, i.e. they could continue whatever selfish behaviour led to the disapproval, without consequence. In such cases, ignoring disapproval would pay better than avoiding disapproval, and disapproval-avoidance would not evolve. It only pays to avoid someone's disapproval if that disapproval is eventually followed by something tangible that *does* affect the offender's fitness (or proxies thereof), such as physical punishment, monetary fines or fewer cooperative interactions because of ostracism, lower trust, less help received, and so on; for simplicity, we collectively refer to all these consequences as ‘costly punishment’ because of the costs they impose on offenders (see Barclay, in press). Thus, disapproval – and fear of disapproval – should only raise cooperation if it is eventually followed by tangible consequences, i.e. if it has ‘teeth’, or has the possibility of doing so (e.g. disapproval by familiar others vs. strangers; Gächter & Fehr, 1999). Toothless disapproval should eventually be ignored: it might raise cooperation in the short term, but will eventually cease to be effective, such that cooperation falls.

Our argument is based on other approaches that view disapproval and threats not as a strict alternative to punishment, but as a complementary precursor to tangible punishment. For example, in her classic studies, Elinor Ostrom (1990) noted that a key design feature of successfully managed commons is a system of escalating forms of sanctioning. In such systems, non-cooperators are first met with disapproval or low-cost punishment, but continued offences incur greater severity and costliness of punishment (and less community support to reintegrate the offender; see Boehm, 1999; Weissner, 2020). Consistent with such field studies, laboratory experiments show that when both disapproval and costly punishment are available, it can result in higher levels of cooperation/group welfare than when only one of these options is available (Noussair & Tucker, 2005; Ostrom et al., 1992); gossip is also much more effective at promoting cooperation when it can be followed by ostracism (Feinberg et al., 2014). Similarly, some evolutionary theories posit that expressions of anger and moral condemnation exist as a threat signal, communicating that costly punishment is likely to follow from that person (e.g. Sell, 2011; Sell et al, 2009). Thus, disapproval and costly punishment can be seen as complementary parts of a process, not as isolated decision alternatives for the disapprover/punisher, nor as psychologically unrelated stimuli for the disapproved/punished.

Our argument about tangible vs. toothless punishment will also be familiar to many parents or teachers. Anecdotally, schoolchildren often learn which teachers are strict enough to be worth listening to, and which teachers’ disapproval can be ignored because they don't follow through with discipline. If something is tempting enough to a child, they may risk their parents’ disapproval if they have learned that disapproval is the only consequence they will face. In short, many children easily learn when they can ignore disapproval, and from whom, vs. when that disapproval is worth attending to because it predicts later consequences. We predict a similar effect with group cooperation: people should eventually ignore ‘toothless’ disapproval, such that cooperation rates eventually decline in the absence of tangible consequences like monetary punishment, but will persist in the presence of such consequences.

More generally, our argument is that humans evolved to be flexible enough to learn about the payoffs of different (un)cooperative strategies. Much work shows that people use payoff-based learning in public goods games, such that they eventually learn to defect when doing so pays best (e.g. Burton-Chellew et al., 2015, 2017; Burton-Chellew & Guerin, 2021); in a meta-analysis of 237 public goods games, Burton-Chellew and West (2021) show that when it is easier to learn that defection pays best, people do so more quickly. In social situations, humans rely on cues of others’ behaviour, like their disapproval, to make inferences about what behaviours will pay off best. However, humans should eventually habituate to uninformative cues about the payoffs for cooperation (Barclay, 2011), just like non-humans habituate to uninformative stimuli of any sort (Domjan & Burkhard, 1993). For example, predators will habituate to the defensive eyespots that some prey species use to mimic a larger organism (Blest, 1957; Edmunds, 1974; Stevens, 2005); humans seem to also habituate to images of eyes, such that if eye images do affect cooperative behaviour (a still-debated question), they only do so temporarily (Sparks & Barclay, 2013; but see Rotella et al 2021). Similarly, (uninformative) reputational cues have only a temporary effect on donations in church (Soetevent, 2005). Relating this back to the current study, we predict that people will initially respond to both tangible and toothless punishment (i.e. monetary sanctions vs. disapproval alone), given that these are normally cues about social payoffs in the real world. However, we predict that participants will eventually habituate to punishment that has no tangible effect on their payoff (i.e. disapproval alone), but will continue to respond to punishment that does have a tangible effect on their payoff (i.e. monetary punishment).

### The present study

The Public Goods Game (PGG) has been widely deployed in experimental social science for examining cooperative behavior across multiple iterations, including the use of variants of the game to allow costly punishment and/or communication of disapproval. The basic PGG can be conceived as an *N*-player expansion of the two-player Prisoner's Dilemma; donating more of one's endowment to the group's public good increases group earnings but decreases individual earnings. We used 4-player groups, a common group size in this literature. (See Methods section for more information.)

Our general prediction is that disapproval will not maintain long-run cooperation, but this offers no precise guidance as to how many rounds of a public goods game should be considered ‘long-run’. We chose to conduct 40 rounds, so that our methods would iterate the game more times than most other public goods experiments, and allow sufficient time for participants to learn to ignore toothless punishment. In their 2021 meta-analysis, Burton-Chellew and West identified 130 papers presenting results from 237 basic public goods game, of which only three studies included more than 30 rounds, representing 1.1% of participants in their aggregated data.

We ran a basic public goods game as a control condition. We included a condition with a round of costly (i.e. monetary) punishment after each basic round, and also a condition with a round of disapproval ratings after each basic round (i.e. numerical ratings of disapproval). To separate *considering disapproval* from the *communication of disapproval*, we included a second control condition, in which participants made the same disapproval ratings without the ratings being communicated to other players. We predicted that cooperation would be higher – and remain higher – in the condition with monetary punishment than in the control condition, but that cooperation would temporarily rise but then fall in the disapproval condition.

## 2. Methods

### 2.1. Participants and recruitment

Participants were 224 undergraduate students (78 male, 146 female, mean age = 18.8 years ± SD 1.5 years) recruited from a pool of psychology students at the University of Guelph in 2012–2013. Participants earned course credit for their participation, plus any monetary earnings from their games (see below); these earnings averaged *M* = $8.25 (SD = $1.55) in 2012 dollars. Participants used an online system to sign up for timeslots to complete the study in a computer lab on campus within sessions of eight people. They were provided with information about the study, gave their informed consent and began the study. They played 40 rounds of public goods games in groups of four (*K* = 56), in a between-subjects design with four conditions (see below). The University of Guelph Research Ethics Board approved the study.

We conducted the study before power analyses were the norm. Instead, our *a priori* stopping rule was to conduct as many sessions as we had participants for in the 2012–2013 academic year, provided we had at least 10 four-person groups in each condition. A *post hoc* power analysis (G*Power 3.1.9.7) shows that with 56 groups, 40 rounds and a correlation of *r* = 0.68 for cooperation between rounds (observed), we would have 84% power to detect a between-groups effect of *f* = 0.40. Predicting that effect size is justified based on the large effects typically seen for punishment, e.g. a meta-analysis by Balliet et al. (2011) reports an average Cohen's *d* = 0.92 for the effect of punishment in experiments with repeated groups.

### 2.2. Materials

The z-tree programs that ran the public goods games and the scripts used to (verbally) inform participants about the study are openly available at https://osf.io/gqfjz/?view_only=None.

Preregistration is not available because the data were collected in 2012 and 2013, before pre-registration was the norm. However, we published the general prediction about habituation to uninformative stimuli before the data were collected (Barclay, 2011, 2012).

### 2.3. Participant anonymity protections

One trained research assistant welcomed participants, situated them at workstations in the lab and conducted the sessions. Experiment sessions included eight participants, divided into two 4-person groups with no obvious means of distinguishing group members within the session. Players sat at individual computer workstations separated by dividers that blocked their view of other players and their screens. Group members were identified in games only by a player number that remained constant throughout the procedure but that could not be linked to the players’ real-life identities. Sessions were conducted in silence, except for the instruction period preceding the games. After the experiment, the experimenter recorded each player's earnings privately from their computer screen and placed the money in an envelope for the participant; the experimenter was unaware of what decisions had led to those earnings. Thus, participants’ decisions were anonymous and their earnings were confidential.

### 2.4. Public goods games – procedure and conditions

Players were endowed with 20 ‘lab dollars’ (L$; convertible to Canadian dollars at L$120:1CAD) at the beginning of each round, and were given the opportunity to contribute any integer amount of their endowment to the public good. The total contributed to the public good was multiplied by 1.6 and evenly distributed among all four group members regardless of their contribution; each L$1 contributed to the public good thus paid L$0.40 to each group member. After each round, players were informed of the contributions and earnings of all group members. At the end of the session, all players were paid their total earnings from all rounds. There were four experimental conditions which differed only in the options available to participants after learning of the other players’ contributions in each round but before the next round started.

In the *Costly Punishment* condition (*k* = 16 groups), players (*n* = 64) were given the opportunity to pay to reduce the earnings of each other player. Every L$1 spent on punishing a player reduced that player's earnings by 10%, up to a total of 100% loss, whereupon players learned the aggregate amount of punishment they received from the other group members. Punishment spending was deducted from the punishing player's earnings; thus the use of punishment was constrained by the players’ earnings for that round.

In the *Public Disapproval* condition (*k* = 14 groups), players (*n* = 56) were offered ‘the opportunity to express dissatisfaction with others by applying dissatisfaction points to them’ (in the verbal instructions, the word used was ‘disapproval’), up to 10 points per target per participant. This condition was designed to be very similar to Costly Punishment, including that players learned the aggregate amount of these points assigned to them by the other three group members. However, unlike the costly punishment condition, these points had no effect on anyone's earnings.

In the first control condition, which we call *Private Disapproval* (*k =* 14 groups), players (*n* = 56) were similarly asked to rate their disapproval, but in this case knowing that these ratings would not be shared with any other players, including the target of the disapproval, nor would it affect anyone's earnings. Because participants never found out whether others (dis)approved of their actions, the (dis)approval could not influence anyone's behaviour.

In the second control condition, *Nothing* (*k =* 12 groups), there was no opportunity for punishment or disapproval ratings; players (*n* = 48) simply proceeded to the next round. As such, this control condition is just a ‘baseline’ public goods game.

### 2.5. Instruction to participants

Before the game began, the research assistant presented the rules, using standard scripts for each condition. The scripts thoroughly explained several examples of the payoffs associated with various contribution levels. (The two groups included in each session were always in the same condition, and received this information at the same time.)

Participants were told that ‘the group as a whole makes the most money when everyone donates their full amount to the group fund’ and that ‘a participant who gives less money than the rest of her group members will finish with more money than them’. Participants were given ample opportunity to ask questions about the rules and procedures (but not strategy) before being asked to remain silent for duration of the game ‘to preserve anonymity’. Participants were not told how many rounds they would play; this was a design feature to reduce endgame effects.

### 2.6. Post-experimental questionnaire

After the games concluded, participants provided information about their gender, age, ethnicity, year in university, university major, university grade point average, political leanings, religiosity, how observed and anonymous they felt and how likely they were to discuss the study. They also responded to open-ended questions about their understanding, how their behaviour and punishment changed over the experiment, and what they thought the experiment was about. These questions were mostly to keep participants busy while the experimenter prepared the cash payouts; we have not analysed them except as basic demographic statistics.

### 2.7. Data and statistical methods

Data exported from z-tree were prepared, analysed and visualized in R REF. Raw data and R scripts are openly available at https://osf.io/gqfjz/?view_only=None.

To compare contributions and punishment between conditions across rounds, we used SPSS 29.0.0.0 to conduct GLM tests with ‘round’ as a within-group variable and ‘condition’ as a between-groups variable. To deal with the interdependence of participants in a group affecting each other, we made groups the unit of analysis (i.e. each group of four people is *N* = 1), as is common in public goods experiments including our previous work (e.g. Barclay, 2004; Pleasant & Barclay, 2018). Although we planned our main comparisons in advance, we analysed them using Tukey highest significant difference *post hoc* tests; the results are similar if we use different *post hoc* tests (see the supplementary information S.2.2, S3.2 and S4.3). In the main text we focus on the comparisons most relevant to our hypotheses; the supplementary information includes tables of all post hoc comparisons of contributions (S2.2), punishment and disapproval (S3.2) and profit (S4.3). To support the ‘new statistics’ of presenting effect sizes and confidence intervals (e.g. Cumming, 2014), we also computed linear mixed models in R using the nlme package REF, with groups entered as having a random intercept term in the models. We used models that collapsed across periods, where condition was the only fixed effect, in order to examine the mean contribution differences by condition.

**Figure 1.**
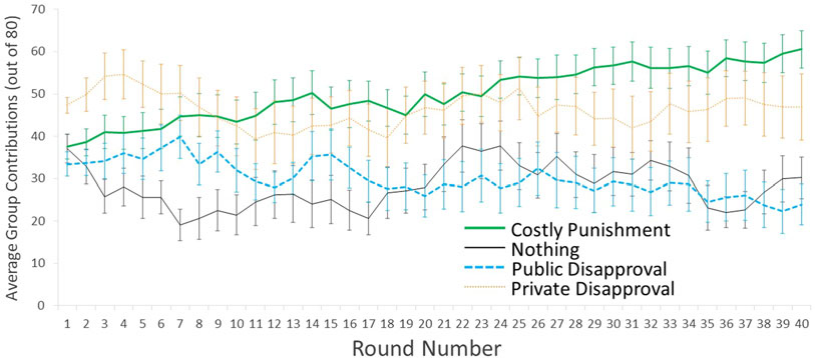
Average contributions (and standard error of the mean, SEM) across 40 rounds of the public goods game with Monetary Punishment (thick solid green line), Public Disapproval (thick dashed blue line), Private Disapproval (thin dotted orange line) and Nothing (thin solid black line). Note: the error bars include variation across groups, but will overestimate variation within a condition over time.

We first computed models for all periods (‘overall’) with ‘period’ as a within-group variable, and we examined the linear and polynomial effects of period. We then did a planned analysis to re-run the same analysis separately for the ‘early’ vs. ‘late’ periods. The seventh round had the highest mean contributions in the Public Disapproval condition (Figure 1), so in Main Text we feature the first seven rounds when discussing ‘early’ effects. This *post hoc* cutoff of seven rounds is maximally generous to the hypothesis that Public Disapproval can compare favourably to Costly Punishment in the short run (i.e. against our hypotheses). For symmetry, we then use the last seven periods when discussing ‘late’ effects. In the supplementary information (S2.3), we provide alternative models based on operationalizing ‘early’ and ‘late’ as 10 or five rounds (our originally planned cutoff); these arbitrary decisions make little difference for interpretation.

## 3. Results

### 3.1. Contributions

A quick glance at Figure 1 shows that contributions increased in the Costly Punishment condition across all 40 rounds, whereas they temporarily increased in the Public Disapproval condition before declining; this matches our predictions. Contributions declined at first in the Nothing condition, as is typical in public goods games (Burton-Chellew & West, 2021), but surprisingly rebounded about halfway through before falling again. Table 1 presents the mean contributions and the linear effect of round, for all rounds combined (1–40), the early rounds (1–7) and the late rounds (rounds 34–40). Figure 1 presents just the overall means and standard error of the mean (SEM) across rounds to facilitate comprehension; in the supplementary information we also present these data with each individual group mean and density plots (S1, S2.1) and individual-level data (S2.4). An omnibus test shows that there were significant differences between conditions in the early rounds, late rounds and overall (all rounds, *F*_3,52_ = 6.22, *p* = 0.001, partial *η*^2^ = 0.26; early rounds only, *F*_3,52_ = 5.75, *p* = 0.002, partial *η*^2^ = 0.25; late rounds only, *F*_3,52_ = 9.55, *p* < 0.001, partial *η*^2^ = 0.36). There were also significant condition × round interactions overall as well as just in the early rounds, but this interaction was not significant in the late rounds (all rounds, *F*_117,2028_ = 2.55, *p* < 0.001, partial *η*^2^ = 0.13; early rounds, *F*_18,312_ = 2.82, *p* < 0.001, partial *η*^2^ = 0.14; late rounds, *F*_18,312_ = 1.25, *p* = 0.22, partial *η*^2^ = 0.07). We compare the individual conditions as follows.

**Table 1.** Summary of means and linear of effect of round on group contributions to the public good

Periods	Condition	Mean percentage contribution [95% CI]	Linear trend beta [95% CI]
All 40	Nothing	35.32 [23.72–46.92]	0.14 [0, 0.27]
	Private Disapproval	57.87 [47.12–68.62]	−0.04 [−0.17, 0.08]
	Public Disapproval	37.62 [26.87–48.37]	−0.35 [−0.44, −0.26]
	Costly Punishment	62.49 [52.43–72.55]	0.66 [0.56, 0.75]
1–7	Nothing	34.66 [24.23–45.09]	−3.07 [−4.06, −2.07]
	Private Disapproval	64.02 [54.35–73.69]	0.31 [−0.92, 1.55]
	Public Disapproval	44.46 [34.8–54.13]	1.2 [0.04, 2.37]
	Costly Punishment	50.98 [41.94–60.03]	1.24 [0.35, 2.13]
34–40	Nothing	33.17 [19.32–47.02]	0.76 [−0.79, 2.3]
	Private Disapproval	59.17 [46.33–72.01]	0.13 [−0.8, 1.07]
	Public Disapproval	31.24 [18.4–44.08]	−0.92 [−1.83, −0.01]
	Costly Punishment	72.38 [60.37–84.39]	0.89 [0.13, 1.65]

Our main prediction was that contributions would be higher with Costly Punishment than with Public Disapproval, especially in the later rounds. Matching this prediction, although there was little difference between these conditions in the early rounds, there was a significant difference in the later rounds and an overall difference across all rounds (early rounds, mean difference = 5.2 ± SE 5.4 [−9.2, 19.7], *p* = 0.77; later rounds, mean difference = 32.9 ± SE 7.2 [13.7, 52.1], *p* < 0.001; all rounds, mean difference = 19.9 ± SE 6.1 [3.7, 36.0], *p* = 0.010). This difference occurred because both conditions showed an increase in contributions in the early rounds (Costly Punishment, *b* = 1.24 [0.35, 2.13]; Public Disapproval, *b* = 1.20 [0.04, 2.37]), but whereas this increase persisted in the Costly Punishment condition (late rounds *b* = 0.89 [0.13, 1.65]; all rounds *b* = 0.66 [0.56, 0.75]), it reversed to become a decline in contributions in the Public Disapproval condition (late rounds *b* = −0.92 [−1.83, −0.01]; all rounds *b* = −0.35 [−0.44, −0.26]). These effects are robust: the trendlines’ 95% confidence intervals do not even overlap in the later rounds or across all 40 rounds (Table 1).

Compared with our main control condition (‘Nothing’), contributions were non-significantly higher in Costly Punishment in the early rounds and significantly higher in the later rounds and overall (early rounds, mean difference = 13.1 ± SE 5.47 [−2.0, 28.1], *p* = 0.11; later rounds, mean difference = 31.4 ± SE 7.6 [11.3, 51.4], *p* < 0.001; all rounds, mean difference = 21.7 ± SE 6.4 [4.9, 38.6], *p* = 0.006). However, Public Disapproval was not as effective compared with Nothing: contributions were slightly but not significantly higher in the Public Disapproval condition in the early rounds, but the two conditions were very similar in the late rounds and overall (early rounds, mean difference = 7.8 ± SE 5.9 [−7.7, 23.4], *p* = 0.54; later rounds, mean difference = −1.5 ± SE 7.8 [−22.2, 19.1], *p* = 1.00; all rounds, mean difference = 1.8 ± SE 6.5 [−15.5, 19.2], *p* = 0.99). In fact, there were periods where contributions were higher in Nothing than in Public Disapproval, including the average of the Late rounds. These results match our corollary main prediction: Costly Punishment was successful at increasing contributions (relative to baseline), but Public Disapproval was only temporarily successful (if that) and was eventually completely ineffective. In fact, Table 1 shows that the confidence intervals do not even come close to overlapping for the linear effects of round in these three conditions.

Surprisingly, contributions started high in the Private Disapproval condition and remained high. In fact, it was the condition with the highest contributions in the early rounds (Private Disapproval vs. Costly Punishment, mean difference = 10.4 ± SE 5.4 [−4.0, 24.9], *p* = 0.24; Private Disapproval vs. Public Disapproval, mean difference = 15.6 ± SE 5.6 [0.7, 30.6], *p* = 0.037; Private Disapproval vs. Nothing, mean difference = 23.5 ± SE 5.9 [7.9, 39.0], *p* = 0.001); these differences appeared from the first round (see the supplementary information S2.2, S2.3.3). Furthermore, contributions did not drop significantly in the Private Disapproval condition (linear trend *b* = −0.04 [−0.17, 0.08]). This was very surprising: disapproval could not possibly have affected others in the Private Disapproval condition, given that the dissatisfaction ratings were not conveyed to any other party, including the target of the dissatisfaction. As such, we suspected a possible type I error. After seeing the between-group variation (supplementary information Figure S1) and individual-level contributions (supplementary information Figure S2.4.4), we conducted a *post hoc* analysis that split the Private Disapproval chronologically into the first seven groups vs. the last seven groups (supplementary information section S2.5). This *post hoc* analysis shows that only the first groups of Private Disapproval had high contributions, whereas the later groups were more similar to Public Disapproval and Nothing (supplementary information Tables S2.5, S2.5.1); in all other conditions the two halves were similar (supplementary information Figures S2.5, S2.5.1). This provides a data-based reason to believe that the abnormally high contributions in Public Disapproval were a statistical fluke in the first several groups that did not replicate in the later groups. We have no other good explanation for why contributions were so high in this condition.

### 3.2. Punishment and disapproval

We had no strong predictions about how the type of punishment or disapproval would affect the amount of each, but we present these data for completeness. We omit the Nothing condition because neither punishment nor disapproval was possible in that condition. In the other three conditions, each of the four group members could assign up to 10 punishment or disapproval points to each of the other three group members, so the maximum points per group was 120.

**Figure 2.**
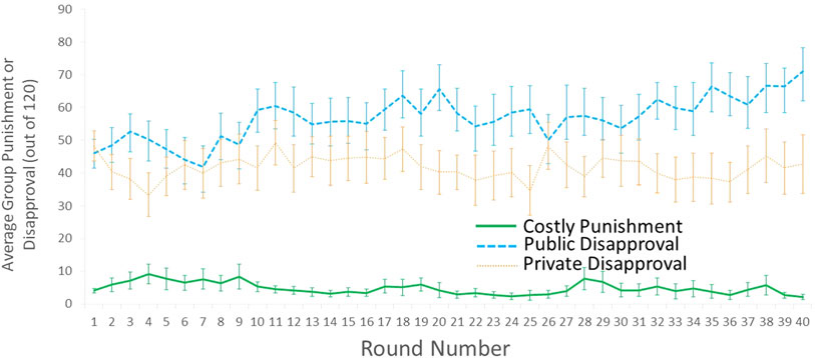
Average punishment or disapproval points (and SEM) across 40 rounds of the public goods game with Monetary Punishment (thick solid green line), Public Disapproval (thick dashed blue line) and Private Disapproval (thin dotted orange line). Note: the error bars include variation across groups, and as such will overestimate variation within a condition over time.

Figure 2 shows the amount of punishment and disapproval points assigned per group; the supplementary information Material (S3.1) gives the means and linear trends overall and in the early and late rounds. There were overall differences across conditions, and there was also a condition × round interaction (main effect of condition: *F*_2,41_ = 37.09, *p* < 0.001, partial *η*^2^ = 0.64; condition × round interaction: *F*_78,1599_ = 1.83, *p* < 0.001, partial *η*^2^ = 0.08; Figure 2). Much of this difference is because there was much less (monetary) punishment in the Costly Punishment condition than there was (costless) disapproval in either the Public or Private Disapproval conditions (Costly Punishment vs. Public Disapproval, mean difference = 52.0 ± SE 6.3 [36.7, 67.2], *p* < 0.001; Costly Punishment vs. Private Disapproval, mean difference = 36.9 ± SE 6.3 [21.7, 52.1], *p* < 0.001). In fact, many groups in Costly Punishment had little punishment, or none in later rounds (supplementary information section S3.3). However, punishment points were arguably on a different scale than disapproval points and are thus not strictly comparable – the maximum punishment receivable by a single person would reduce their earnings by an impossible 300%. Instead, we can only directly compare magnitudes in the two disapproval conditions, or the trends over time in all three conditions.

There was marginally more Public Disapproval than Private Disapproval (mean difference = 15.0 ± SE 7.9 [−0.7, 30.8], *p* = 0.063) and a significant condition × round interaction between these conditions (*F*_39,1014_ = 1.56, *p* < 0.017, partial *η*^2^ = 0.06) which was driven by significantly different linear trends (*F*_1,26_ = 4.77, *p* = 0.038, partial *η*^2^ = 0.16). Disapproval increased over time in the Public Disapproval condition but not in the Private Disapproval condition or Costly Punishment condition (linear trend in Public Disapproval, *F*_1,13_ = 13.69, *p* = 0.003, partial *η*^2^ = 0.51; linear trend in Private Disapproval, *F*_1,13_ = 0.03, *p* = 0.85, partial *η*^2^ = 0.00; linear trend in Costly Punishment, *F*_1,15_ = 2.09, *p* = 0.17, partial *η*^2^ = 0.12). The different trends for disapproval are most likely because contributions decreased over time in the Public but not Private Disapproval condition (see Section 3.1).

**Figure 3.**
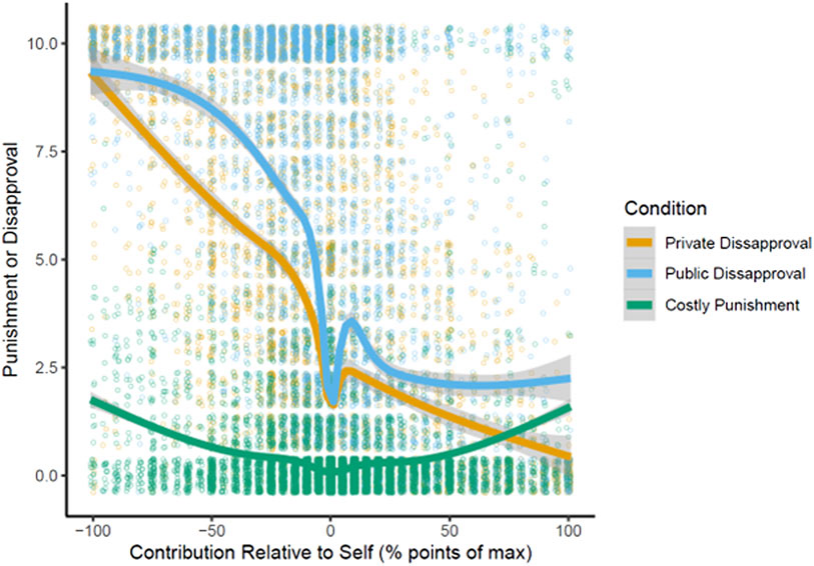
Player-level data on negative reactions to other players as a function of how much more or less that other person contributed relative to oneself. Each point represents one of the three individual punishment or disapproval decisions each player makes in each round (i.e. one for each other group member), with jitter. Curves are smoothed using Generalized Additive Models; the grey shaded areas are 95% confidence intervals of the curve.

Figure 3 shows that most disapproval is directed towards low contributors, as is typical in punishment experiments (see supplementary information S3.4 for a breakdown by early and late rounds). However, there is also some punishment and disapproval targeting high contributors (‘antisocial punishment’, Herrmann et al., 2008; Pleasant & Barclay, 2018; ‘do-gooder derogation’, Monin, 2007). The jittered individual-level view reveals what we call the ‘Hypocrisy Valley’ – near-identical contributions receive very low disapproval compared with the rest of the curve; this appears most pronounced with Public Disapproval. Supplementary information Figure S3.5 shows that the more punishment or disapproval people receive, the more they change their behaviour in the subsequent round. However, this effect is less pronounced with Public Disapproval than with Private Disapproval, suggesting that it is not the disapproval itself that causes the increased contributions (for discussion, see Barclay, submitted; Raihani & Bshary 2019); in fact, receiving public disapproval might make that disapproval less effective.

### 3.3. Profits

**Figure 4.**
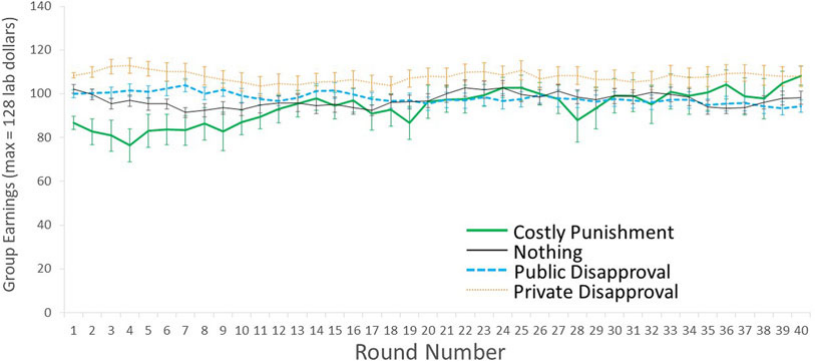
Average group earnings (and SEM) across 40 rounds of the public goods game with Monetary Punishment (thick solid green line), Public Disapproval (thick dashed blue line), Private Disapproval (thin dotted orange line) and Nothing (thin solid black line). Note: the error bars include variation across groups, and as such will overestimate variation within a condition over time.

Costly Punishment reduces the earnings of both the punisher and the target. As a result, group earnings were lower in the early rounds in the Costly Punishment condition than in the Nothing, Public Disapproval and Private Disapproval conditions (*F*_3,52_ = 9.96, *p* < 0.001, partial *η*^2^ = 0.37, mean differences = 14.2, 19.0, and 28.3, SE = 5.6, 5.3 and 5.3, 95% CI [−0.5, 29.0], [4.8, 33.1] and [14.2, 42.5], *p*s = 0.062, 0.004 and <0.001, respectively, see Figures 4 & S4.2, Table S4.1). There were no significant group differences in early earnings among the latter three conditions, although Private Disapproval had marginally higher earnings than Nothing (see supplementary information S4.3 for all other comparisons).

However, given that Costly Punishment also increases group contributions (section 3.1), the cost of punishment might only be temporary. Indeed, there were significantly different patterns of earnings across rounds (round X condition interaction: *F*_117,2028_ = 2.73, *p* < 0.001, partial *η*^2^ = 0.14), which were driven by different linear patterns (*F*_3.52_ = 8.53, *p* < 0.001, partial *η*^2^ = 0.33, Figure 4). More specifically, the Costly Punishment condition showed a significant linear increase in earnings over time (*F*_1,15_ = 13.31, *p* = 0.002, partial *η*^2^ = 0.74), whereas there was no linear change in earnings in the Nothing and Private Disapproval conditions, and there was a linear decrease in earnings in the Public Disapproval condition (Nothing,^1^ *F*_1,11_ = 0.50, *p* = 0.49, partial *η*^2^ = 0.04; Private Disapproval, *F*_1,13_ = 0.41, *p* = 0.84, partial *η*^2^ = 0.00; Public Disapproval, *F*_1,13_ = 9.67, *p* = 0.008, partial *η*^2^ = 0.43).

As a result of these different linear patterns, the four conditions did not differ in earnings in the late rounds (*F*_3,52_ = 1.71, *p* = 0.18, partial *η*^2^ = 0.09). Earnings were slightly but not significantly higher in Costly Punishment than in Nothing or Public Disapproval (mean differences = 6.1 and 7.0, SE 6.7 and 6.4, 95% CI [−11.8, 23.9] and [−10.1, 24.1], *p* = 0.80 and 0.70, respectively; see supplementary information for other comparisons). This suggests that if the game had continued longer, then costly punishment might have eventually had higher earnings than these conditions (as per Gächter et al., 2008), but the non-significant results in the late rounds preclude any conclusions about this.

## 4. Discussion

### 4.1. Primary hypotheses: the long-term effectiveness of punishment vs. disapproval

The results support our main hypotheses: cooperation increased at first when participants could either communicate their disapproval or pay to punish each other, and it increased to a similar extent in both conditions. However, as predicted, communicated disapproval was not as effective in the long run – public goods contributions continued to increase when punishment was possible, but eventually decreased when only disapproval was possible. In fact, disapproval eventually became completely ineffective, in that participants eventually contributed the same or less to the public good when disapproval could be communicated than in a baseline public goods with no disapproval or punishment (Nothing condition).

These findings speak against the idea that ‘disapproval avoidance’ alone is enough to incentivize long-term cooperation, contrary to what some researchers have implied (e.g. Brook & Servátka, 2016) Fehr & Falk, 2002; Festré & Garrouste, 2014; López-Pérez & Vorsatz, 2010; Masclet et al., 2003, 2013; Xiao & Houser, 2009). People may be motivated to avoid disapproval in the short term – cooperation did initially increase in the Public Disapproval condition – but eventually, this communication of disapproval became ineffective as it carried no consequences. Our advice for would-be social engineers is to not rely solely on disapproval or negative feedback, like verbal scoldings, frowning emoticons or comparing their cooperative behaviour unfavourably with that of others (e.g. feedback on energy bills comparing their usage with the average; Allcott, 2011; Ayres et al., 2013; Schulz et al., 2007). Our results suggest that disapproval-based manipulations will eventually become ineffective unless they are (at least occasionally) accompanied by sanctions that matter. That being said, disapproval-based manipulations could have a lasting effect if they cause people to invest in long-lasting changes, such as if disapproval about someone's energy use causes them to buy energy-efficient appliances or learn new habits; those investments may still function after the person starts ignoring the (dis)approval.

So why are people averse to disapproval if they eventually come to ignore it? Outside the laboratory, disapproval usually does eventually carry consequences, including lower trust, the loss of valued partnerships, fewer new partnerships, physical punishment or formal sanctions (for reviews, see Barclay, 2013, in press). Disapproval is cheap and easy to implement, so it functions as a warning that these tangible consequences will follow if the target continues to transgress without making amends (see also graded punishment, Ostrom, 1990). As such, humans probably evolved to dislike disapproval because of those eventual social consequences. However, humans also evolved to be flexible and to learn from consequences – or lack thereof – which allows them to eventually ignore warnings about threats that never materialize, including disapproval. This also protects them from manipulation by others who would use disapproval as a cheap threat which is never intended to be carried out – eventually people will only respond to disapproval from people whose disapproval carries consequences. As such, this learning should be largely person- or context-specific: people should become averse to disapproval from people whose disapproval carries consequences, ignore disapproval from people who are nothing but cheap talk and generalize across people based on base rates and how similar a given person is to known others (e.g. is this new person more similar to known ‘tough people’ or ‘cheap talkers’).

### 4.2. Profits

Our results show that because of the costs of sanctions, costly punishment reduces group profits at first, but this disadvantage disappears over time. By the end of 40 rounds, profits were slightly but non-significantly higher in the Costly Punishment condition than in Public Disapproval or Nothing, even after subtracting the cost of punishment. Other research suggests that the most efficient system is one that allows both disapproval and punishment, so that disapproval can be used as a cheap warning which is backed up by the presence of costly punishment when needed (Noussair & Tucker, 2005; Ostrom, 1990). Future research should explicitly compare the long-term profits when there is punishment alone vs. disapproval alone vs. punishment and disapproval together.

### 4.3. Surprising results: private disapproval, cooperative rebounds, within-person variation

Our most surprising result is the high cooperation rates in the Private Disapproval condition. In this condition, participants rated their disapproval of others’ actions, but those ratings were not communicated to anyone and could thus have no effect on others. This was intended to be a control condition, where we expected cooperation to be similar to the baseline Nothing condition. However, it started with significantly higher cooperation than any other condition (supplementary information S2.3.3) and showed no long-term decline (Table 1).

We suspected that these high contributions in Private Disapproval were a type I error. To test this, we did a *post hoc* analysis where we split each condition in half chronologically, to separate the first several groups from the later several groups (see supplementary information section S2.5). This chronological split shows that contributions were only high in the first few groups of Private Disapproval, but this pattern did not replicate in the later several groups; the later groups of Private Disapproval were statistically very similar to the Public Disapproval and Nothing conditions (supplementary information section S2.5). In contrast, the other three conditions were consistent between the chronologically earlier and later groups, i.e. the other three conditions did replicate. This gives a data-driven reason to believe that the high contributions in Private Disapproval were a type I error driven by the first several sessions in that condition, given that the high contributions were not replicated in the later several sessions.

If it is indeed a real effect, we have no good explanation for it, and can only make *post hoc* speculations on its causes. For example, perhaps contributions remain high when people can privately let their emotions out (‘venting’), because they do that instead of dropping their contributions, and private venting does not provoke a negative reaction from others (see discussion in the supplementary information S3.5). However, contributions are higher in the Private Disapproval condition from the very first round, which suggests that it is not just about the process – either there is an anticipation effect, or it is a type I error. We recommend that future research attempt to replicate the effect of Private Disapproval before relying on what we have reason to believe is a false positive result.

Another surprising result was the rebound in contributions in the baseline Nothing condition. Contributions dropped at first, as they do in typical public goods games (for a review, see Burton-Chellew & West, 2021). However, contributions spontaneously recovered about half-way through the Nothing condition (around round 20), even reaching their initial levels before slowly declining again. We do not know why – perhaps some participants got bored with multiple rounds of low contributions and tried to liven it up with spontaneously high contributions (see individual-level contributions in the supplementary information Figure S2.4.1). It does not appear to be a fluke: the same pattern is independently seen in both the first and last few groups of Nothing (i.e. splitting the Nothing condition in half chronologically; supplementary information S2.5). Very few experiments use as many rounds as we did, so perhaps this recovery would occur more often if researchers tested for it.

A final surprising result was the amount of variation within the same individuals across rounds (see supplementary information S2.4, S3.6). For example, some individuals repeatedly alternated between full and zero contributions, which probably contributed to the variation between groups within a condition (supplementary information S1) and may have contributed to the recovery of cooperation in the Nothing condition. This incidental finding speaks against the idea that participants can be cleanly classified as types like ‘cooperator’ and ‘defector’ (e.g. Peysakhovich et al., 2014). We encourage future researchers to examine the causes of such within-individual variation.

### 4.4. Limitations and conclusions

Our research has some limitations that warrant mention. First, the participants were all students at a Canadian university, which means they may not be representative of all ages, economic classes or cultures. However, our main effect – habituation to inconsequential disapproval – will probably generalize to other groups, given that habituation is well established in humans and non-humans (e.g. Domjan & Burkhard 1993).

Second, we used one particular laboratory method – a public goods game – to test our hypotheses. This gave us greater control over the manipulation, but sacrifices some external validity, such that the results might not generalize to other social scenarios or incentive structures. Ultimately though, we are examining incentives, so we predict that these results will generalize to any social scenarios with similar incentives, i.e. when the private incentives for some behaviour (e.g. selfishness) are opposed by social or legal pressures to not do that behaviour (e.g. through punishment, disapproval, fines, criminal charges). Future studies should test for habituation to ‘toothless’ disapproval outside the laboratory in real-life social situations.

Third, we only tested one type of tangible punishment (monetary fines) and one type of ‘toothless’ disapproval (ratings on a computer). Other kinds of punishment and disapproval might elicit shorter or longer lasting cooperation. In fact, our hypothesis explicitly predicts variation in the effect size: there will be less habituation when disapproval is more consequential, with ‘consequences’ defined as the impact on proxies for evolutionary fitness (e.g. wealth, status, prestige, social support). Similarly, people should learn to ignore inconsequential disapproval more quickly when the behaviour it incentivizes is more costly (e.g. a heavy vs. light sacrifice), and when it is clearer that the disapproval has few impactful consequences. Furthermore, as one reviewer suggests, it may be easier to learn to ignore abstract disapproval like numerical ratings than real-life expression of disapproval through angry words or facial expressions; the latter involve unlearning or overriding common cues that normally predict consequences from that person. Ultimately, we predict that the easier it is to learn that disapproval is ‘toothless’, the faster that people will come to ignore it.

Overall, our findings support the general hypothesis that humans use cues in their environment to predict the consequences of their actions, but will eventually learn to ignore uninformative cues (i.e. those that do not predict any tangible consequences). These findings also support the use of graduated sanctions: in the short term, warnings and disapproval might be cheap alternatives to tangible punishments like fines or lost trust, but they must eventually be followed by consequential punishments, lest the disapproval lose its effectiveness. Would-be social engineers would be unwise to rely solely on disapproval or social norms, unless norm-breakers suffer tangible consequences – it is those tangible consequences that give people a reason to care about normative approval.

## Supporting information

Sparks et al. supplementary material Sparks et al. supplementary material

## Footnotes

1 Earnings in the Nothing condition rebounded with the unexpected increase in contributions midway (Section 3.1), resulting in significant cubic and quartic trends (*F*_1,11_ = 5.05 and 6.35, *p* = 0.046 and 0.028, partial *η*^2^ = 0.32 and 0.37, respectively). No other conditions showed significant quadratic, cubic, quartic or quintic trends (all *p* > 0.1); any polynomial trends at higher orders are probably due to overfitting and will not be discussed further.

## Supplementary material

To view supplementary material for this article, please visit https://doi.org/10.1017/ehs.2024.41

## Research transparency and reproducibility

Raw data and R scripts are openly available at https://osf.io/gqfjz/?view_only=None
