# Supplementary material for: Expressed disapproval does not sustain long-term cooperation as effectively as costly punishment
Source: Evol Hum Sci. 2024 Dec 26;6:e53. doi: 10.1017/ehs.2024.41 (PMC11706683; doi:10.1017/ehs.2024.41)
Supplement: Sparks et al. supplementary material [file S2513843X24000410sup001.docx]

Supplementary Materials for:

Mere disapproval does not sustain cooperation as effectively as costly punishment in a 40-round public goods game

Adam Sparks, Tyler Burleigh, and Pat Barclay*

University of Guelph

*Corresponding Author

Pat Barclay, Department of Psychology, University of Guelph, Guelph, ON, Canada

1-519-824-4120 ext. 58247, [barclayp@uoguelph.ca](mailto:barclayp@uoguelph.ca), [www.patbarclay.com](http://www.patbarclay.com)

This Supplementary Material includes:

**S1: Graphs of mean session-level data and density plots 2**

**S2: Supplementary information on contributions 3**

S2.1 Graphs of session-level data & density plots of contributions in early vs. late rounds 3

S2.2 Post hoc tests of between-condition differences in contributions 4

*S2.2.1 Post hoc tests of all 40 rounds 4*

*S2.2.2 Post hoc tests of early rounds 6*

*S2.2.3 Post hoc tests of late rounds 8*

S2.3 Contributions in “early” & “late” rounds, after redefining “early” and “late” 10

S2.3.1 “Early” & “late” as the first & last 5 rounds (rounds 1-5 vs. 36-40) 10

S2.3.2 “Early” & “late” as the first & last 10 rounds (rounds 1-10 vs. 31-40) 11

S2.3.3 “Early” & “late” as the first & last 1 round (round 1 vs. 40) 12

S2.4 Graphs of individual-level contributions data 13

S2.5 Contributions in the 1^st^ vs. 2^nd^ half of the groups (chronologically) 16

S2.5.1 Supplement About the First & Second Chronological Halves of the Sessions 18

**S3: Supplementary Information on Punishment & Disapproval 22**

S3.1 Table of means and trends of punishment & disapproval 22

S3.2 Post hoc tests of between-condition differences in punishment/disapproval 23

*S3.2.1 Post hoc tests of all 40 rounds 23*

*S3.2.2 Post hoc tests of early rounds 24*

*S3.2.3 Post hoc tests of late rounds 25*

S3.3 Closer visualization of costly punishment 26

S3.4 Punishment received in early vs. late rounds 28

S3.5 Effects of receiving punishment and disapproval 29

S3.6 Graphs of individual-level punishment and disapproval data 31

**S4: Supplementary Information on Profits 33**

S4.1 Table of means and trends of profits 33

S4.2 Graphs of session-level profits & density plots in early vs. late rounds 33

S4.3 Post hoc comparisons of profits in early rounds, late rounds, and overall 34

*S4.3.1 Post hoc tests of all 40 rounds 34*

*S4.3.2 Post hoc tests of early rounds 36*

*S4.3.3 Post hoc tests of late rounds 38*

# S1 Graphs of Mean Session-Level Data and Density Plots


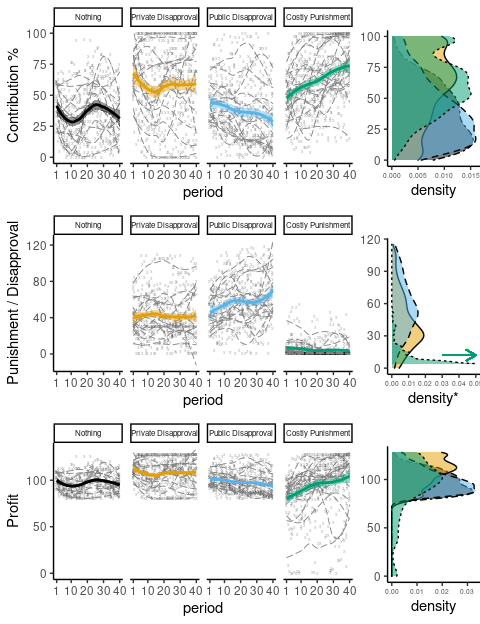


Figure S1: Public goods contributions, punishment & dissatisfaction ratings, and overall profits, separated by condition with individual group means and density plots. In the scatterplots, each ‘x’ is a group’s total contribution, punishment/disapproval, or profit in a period; the colored solid line and shaded 95% confidence intervals summarize period mean trends within each condition using LOESS smoothing; the grey dashed lines summarize group mean trends with LOESS. Density plots summarize these data, collapsing across period ranges within condition. The density range for Punishment / Disapproval is truncated to focus on the disapproval condition ranges; densities at near-zero levels of costly punishment peak around 0.20 density (see S3.3.2).

# S2 Supplementary Information on Contributions

## S2.1 Graphs of Session-Level Data on Contributions in Early and Late Rounds


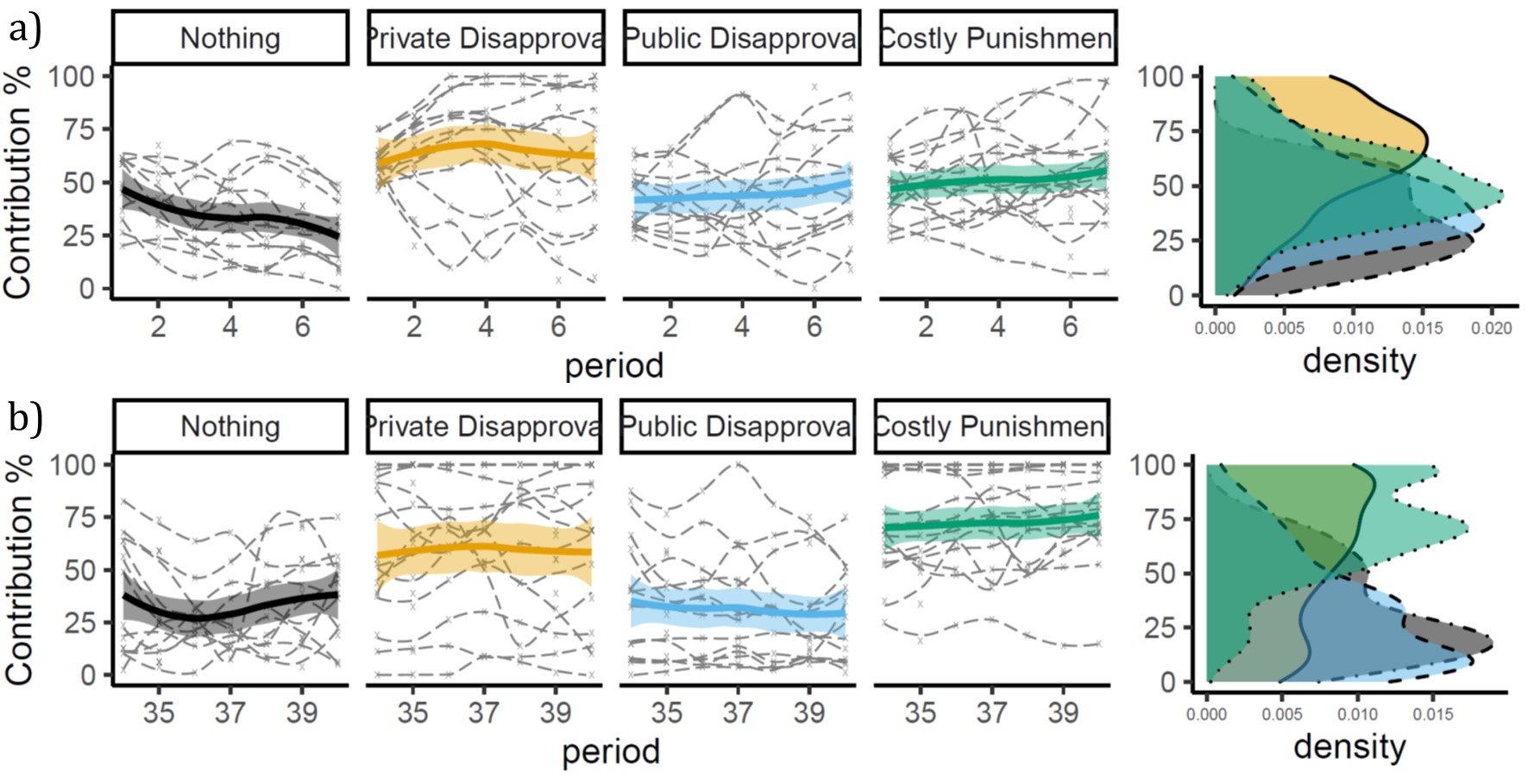


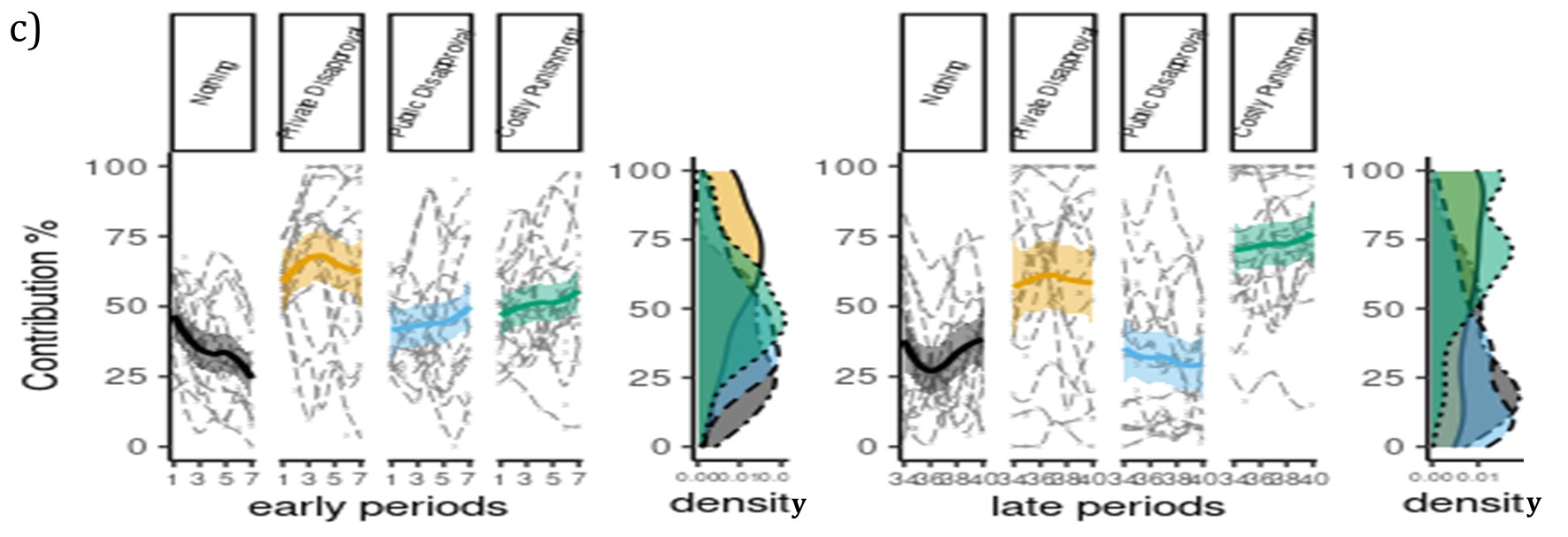


Figure S2.1: Detailed comparison of public goods contributions with 95% confidence intervals in a) early periods (rounds 1-7), b) late periods (rounds 34-40), c) both early and late on the same row for easier visual comparison. In the scatterplots, each ‘x’ is a group’s total contribution; the colored solid lines and shaded 95% confidence intervals summarize period mean trends within each condition using LOESS smoothing; the grey dashed lines summarize group mean trends with LOESS. Density plots summarize these data, collapsing across period ranges within each condition.

## S2.2 Post Hoc Tests of Between-Condition Differences in Contributions

The main text focuses primarily on our planned comparisons of contributions between Costly Punishment vs. Public Disapproval, Costly Punishment vs. Nothing, and Public Disapproval vs. Nothing, as these were the most important comparisons for our hypotheses. Here we present post hoc comparisons of earnings per round in all conditions using Tukey HSD, Scheffe, and Sidak tests from our Repeated Measures GLM in SPSS with the four between-subjects conditions. The results are very similar with all three types of post hoc tests.

### **S2.2.1 Post Hoc Tests of Contributions in All 40 Rounds in All Conditions**

*Table S2.2.1 Post hoc tests of contributions in all 40 rounds*

| **Multiple Comparisons** | | | | | | | |
| --- | --- | --- | --- | --- | --- | --- | --- |
| Measure: MEASURE_1 | | | | | | | |
|  | (I) condition | (J) condition | Mean Difference (I-J) | Std. Error | Sig. | 95% Confidence Interval | |
|  |  |  |  |  |  | Lower Bound | Upper Bound |
| Tukey HSD | CostPun | NoDis | 21.7359^*^ | 6.35009 | .006 | 4.8822 | 38.5897 |
|  |  | PrivDis | 3.6975 | 6.08539 | .929 | -12.4537 | 19.8488 |
|  |  | PubDis | 19.8940^*^ | 6.08539 | .010 | 3.7428 | 36.0452 |
|  | NoDis | CostPun | -21.7359^*^ | 6.35009 | .006 | -38.5897 | -4.8822 |
|  |  | PrivDis | -18.0384^*^ | 6.54160 | .039 | -35.4004 | -.6764 |
|  |  | PubDis | -1.8420 | 6.54160 | .992 | -19.2040 | 15.5201 |
|  | PrivDis | CostPun | -3.6975 | 6.08539 | .929 | -19.8488 | 12.4537 |
|  |  | NoDis | 18.0384^*^ | 6.54160 | .039 | .6764 | 35.4004 |
|  |  | PubDis | 16.1964 | 6.28496 | .060 | -.4845 | 32.8773 |
|  | PubDis | CostPun | -19.8940^*^ | 6.08539 | .010 | -36.0452 | -3.7428 |
|  |  | NoDis | 1.8420 | 6.54160 | .992 | -15.5201 | 19.2040 |
|  |  | PrivDis | -16.1964 | 6.28496 | .060 | -32.8773 | .4845 |
| Scheffe | CostPun | NoDis | 21.7359^*^ | 6.35009 | .014 | 3.3889 | 40.0830 |
|  |  | PrivDis | 3.6975 | 6.08539 | .946 | -13.8847 | 21.2798 |
|  |  | PubDis | 19.8940^*^ | 6.08539 | .020 | 2.3117 | 37.4762 |
|  | NoDis | CostPun | -21.7359^*^ | 6.35009 | .014 | -40.0830 | -3.3889 |
|  |  | PrivDis | -18.0384 | 6.54160 | .067 | -36.9387 | .8620 |
|  |  | PubDis | -1.8420 | 6.54160 | .994 | -20.7423 | 17.0584 |
|  | PrivDis | CostPun | -3.6975 | 6.08539 | .946 | -21.2798 | 13.8847 |
|  |  | NoDis | 18.0384 | 6.54160 | .067 | -.8620 | 36.9387 |
|  |  | PubDis | 16.1964 | 6.28496 | .098 | -1.9624 | 34.3553 |
|  | PubDis | CostPun | -19.8940^*^ | 6.08539 | .020 | -37.4762 | -2.3117 |
|  |  | NoDis | 1.8420 | 6.54160 | .994 | -17.0584 | 20.7423 |
|  |  | PrivDis | -16.1964 | 6.28496 | .098 | -34.3553 | 1.9624 |
| Sidak | CostPun | NoDis | 21.7359^*^ | 6.35009 | .007 | 4.3689 | 39.1030 |
|  |  | PrivDis | 3.6975 | 6.08539 | .991 | -12.9456 | 20.3407 |
|  |  | PubDis | 19.8940^*^ | 6.08539 | .011 | 3.2509 | 36.5371 |
|  | NoDis | CostPun | -21.7359^*^ | 6.35009 | .007 | -39.1030 | -4.3689 |
|  |  | PrivDis | -18.0384^*^ | 6.54160 | .047 | -35.9292 | -.1476 |
|  |  | PubDis | -1.8420 | 6.54160 | 1.000 | -19.7328 | 16.0488 |
|  | PrivDis | CostPun | -3.6975 | 6.08539 | .991 | -20.3407 | 12.9456 |
|  |  | NoDis | 18.0384^*^ | 6.54160 | .047 | .1476 | 35.9292 |
|  |  | PubDis | 16.1964 | 6.28496 | .075 | -.9925 | 33.3854 |
|  | PubDis | CostPun | -19.8940^*^ | 6.08539 | .011 | -36.5371 | -3.2509 |
|  |  | NoDis | 1.8420 | 6.54160 | 1.000 | -16.0488 | 19.7328 |
|  |  | PrivDis | -16.1964 | 6.28496 | .075 | -33.3854 | .9925 |
| Based on observed means.  The error term is Mean Square(Error) = 276.505. | | | | | | | |
| *. The mean difference is significant at the .05 level. | | | | | | | |

CostPun = Costly Punishment

NoDis = Nothing (i.e., No Disapproval or Punishment)

PrivDis = Private Disapproval

PubDis = Public Disapproval

### **S2.2.2 Post Hoc Tests of Contributions in Early Rounds (i.e., Rounds 1-7) in All Conditions**

*Table S2.2.2 Post hoc tests of contributions in the early rounds (rounds 1-7)*

| **Multiple Comparisons** | | | | | | | | |
| --- | --- | --- | --- | --- | --- | --- | --- | --- |
| Measure: MEASURE_1 | | | | | | | | |
|  | (I) condition | (J) condition | Mean Difference (I-J) | Std. Error | Sig. | 95% Confidence Interval | |  |
|  |  |  |  |  |  | Lower Bound | Upper Bound |  |
| Tukey HSD | CostPun | NoDis | 13.0595 | 5.68603 | .112 | -2.0318 | 28.1508 |  |
|  |  | PrivDis | -10.4286 | 5.44901 | .235 | -24.8908 | 4.0336 |  |
|  |  | PubDis | 5.2143 | 5.44901 | .774 | -9.2479 | 19.6765 |  |
|  | NoDis | CostPun | -13.0595 | 5.68603 | .112 | -28.1508 | 2.0318 |  |
|  |  | PrivDis | -23.4881^*^ | 5.85751 | .001 | -39.0345 | -7.9417 |  |
|  |  | PubDis | -7.8452 | 5.85751 | .543 | -23.3916 | 7.7012 |  |
|  | PrivDis | CostPun | 10.4286 | 5.44901 | .235 | -4.0336 | 24.8908 |  |
|  |  | NoDis | 23.4881^*^ | 5.85751 | .001 | 7.9417 | 39.0345 |  |
|  |  | PubDis | 15.6429^*^ | 5.62771 | .037 | .7064 | 30.5794 |  |
|  | PubDis | CostPun | -5.2143 | 5.44901 | .774 | -19.6765 | 9.2479 |  |
|  |  | NoDis | 7.8452 | 5.85751 | .543 | -7.7012 | 23.3916 |  |
|  |  | PrivDis | -15.6429^*^ | 5.62771 | .037 | -30.5794 | -.7064 |  |
| Scheffe | CostPun | NoDis | 13.0595 | 5.68603 | .167 | -3.3689 | 29.4879 |  |
|  |  | PrivDis | -10.4286 | 5.44901 | .311 | -26.1722 | 5.3150 |  |
|  |  | PubDis | 5.2143 | 5.44901 | .821 | -10.5293 | 20.9579 |  |
|  | NoDis | CostPun | -13.0595 | 5.68603 | .167 | -29.4879 | 3.3689 |  |
|  |  | PrivDis | -23.4881^*^ | 5.85751 | .003 | -40.4119 | -6.5643 |  |
|  |  | PubDis | -7.8452 | 5.85751 | .619 | -24.7691 | 9.0786 |  |
|  | PrivDis | CostPun | 10.4286 | 5.44901 | .311 | -5.3150 | 26.1722 |  |
|  |  | NoDis | 23.4881^*^ | 5.85751 | .003 | 6.5643 | 40.4119 |  |
|  |  | PubDis | 15.6429 | 5.62771 | .064 | -.6170 | 31.9028 |  |
|  | PubDis | CostPun | -5.2143 | 5.44901 | .821 | -20.9579 | 10.5293 |  |
|  |  | NoDis | 7.8452 | 5.85751 | .619 | -9.0786 | 24.7691 |  |
|  |  | PrivDis | -15.6429 | 5.62771 | .064 | -31.9028 | .6170 |  |
| Sidak | CostPun | NoDis | 13.0595 | 5.68603 | .145 | -2.4914 | 28.6104 |  |
|  |  | PrivDis | -10.4286 | 5.44901 | .315 | -25.3312 | 4.4741 |  |
|  |  | PubDis | 5.2143 | 5.44901 | .920 | -9.6884 | 20.1169 |  |
|  | NoDis | CostPun | -13.0595 | 5.68603 | .145 | -28.6104 | 2.4914 |  |
|  |  | PrivDis | -23.4881^*^ | 5.85751 | .001 | -39.5080 | -7.4682 |  |
|  |  | PubDis | -7.8452 | 5.85751 | .710 | -23.8651 | 8.1746 |  |
|  | PrivDis | CostPun | 10.4286 | 5.44901 | .315 | -4.4741 | 25.3312 |  |
|  |  | NoDis | 23.4881^*^ | 5.85751 | .001 | 7.4682 | 39.5080 |  |
|  |  | PubDis | 15.6429^*^ | 5.62771 | .044 | .2515 | 31.0343 |  |
|  | PubDis | CostPun | -5.2143 | 5.44901 | .920 | -20.1169 | 9.6884 |  |
|  |  | NoDis | 7.8452 | 5.85751 | .710 | -8.1746 | 23.8651 |  |
|  |  | PrivDis | -15.6429^*^ | 5.62771 | .044 | -31.0343 | -.2515 |  |
| Based on observed means.  The error term is Mean Square(Error) = 221.698. | | | | | | | | |
| *. The mean difference is significant at the .05 level. | | | | | | | | |

CostPun = Costly Punishment

NoDis = Nothing (i.e., No Disapproval or Punishment)

PrivDis = Private Disapproval

PubDis = Public Disapproval

### **S2.2.3 Post Hoc Tests of Contributions in Late Rounds (i.e., Rounds 34-40) in All Conditions**

*Table S2.2.3 Post hoc tests of contributions in the late rounds (rounds 34-40)*

| **Multiple Comparisons** | | | | | | | |
| --- | --- | --- | --- | --- | --- | --- | --- |
| Measure: MEASURE_1 | | | | | | | |
|  | (I) condition | (J) condition | Mean Difference (I-J) | Std. Error | Sig. | 95% Confidence Interval | |
|  |  |  |  |  |  | Lower Bound | Upper Bound |
| Tukey HSD | CostPun | NoDis | 31.3661^*^ | 7.55110 | <.001 | 11.3247 | 51.4074 |
|  |  | PrivDis | 10.5651 | 7.23633 | .469 | -8.6409 | 29.7710 |
|  |  | PubDis | 32.9120^*^ | 7.23633 | <.001 | 13.7061 | 52.1179 |
|  | NoDis | CostPun | -31.3661^*^ | 7.55110 | <.001 | -51.4074 | -11.3247 |
|  |  | PrivDis | -20.8010^*^ | 7.77882 | .048 | -41.4468 | -.1553 |
|  |  | PubDis | 1.5459 | 7.77882 | .997 | -19.0998 | 22.1917 |
|  | PrivDis | CostPun | -10.5651 | 7.23633 | .469 | -29.7710 | 8.6409 |
|  |  | NoDis | 20.8010^*^ | 7.77882 | .048 | .1553 | 41.4468 |
|  |  | PubDis | 22.3469^*^ | 7.47365 | .021 | 2.5111 | 42.1827 |
|  | PubDis | CostPun | -32.9120^*^ | 7.23633 | <.001 | -52.1179 | -13.7061 |
|  |  | NoDis | -1.5459 | 7.77882 | .997 | -22.1917 | 19.0998 |
|  |  | PrivDis | -22.3469^*^ | 7.47365 | .021 | -42.1827 | -2.5111 |
| Scheffe | CostPun | NoDis | 31.3661^*^ | 7.55110 | .002 | 9.5490 | 53.1831 |
|  |  | PrivDis | 10.5651 | 7.23633 | .550 | -10.3426 | 31.4727 |
|  |  | PubDis | 32.9120^*^ | 7.23633 | <.001 | 12.0044 | 53.8196 |
|  | NoDis | CostPun | -31.3661^*^ | 7.55110 | .002 | -53.1831 | -9.5490 |
|  |  | PrivDis | -20.8010 | 7.77882 | .080 | -43.2760 | 1.6740 |
|  |  | PubDis | 1.5459 | 7.77882 | .998 | -20.9291 | 24.0209 |
|  | PrivDis | CostPun | -10.5651 | 7.23633 | .550 | -31.4727 | 10.3426 |
|  |  | NoDis | 20.8010 | 7.77882 | .080 | -1.6740 | 43.2760 |
|  |  | PubDis | 22.3469^*^ | 7.47365 | .040 | .7536 | 43.9402 |
|  | PubDis | CostPun | -32.9120^*^ | 7.23633 | <.001 | -53.8196 | -12.0044 |
|  |  | NoDis | -1.5459 | 7.77882 | .998 | -24.0209 | 20.9291 |
|  |  | PrivDis | -22.3469^*^ | 7.47365 | .040 | -43.9402 | -.7536 |
| Sidak | CostPun | NoDis | 31.3661^*^ | 7.55110 | <.001 | 10.7143 | 52.0178 |
|  |  | PrivDis | 10.5651 | 7.23633 | .624 | -9.2258 | 30.3559 |
|  |  | PubDis | 32.9120^*^ | 7.23633 | <.001 | 13.1211 | 52.7029 |
|  | NoDis | CostPun | -31.3661^*^ | 7.55110 | <.001 | -52.0178 | -10.7143 |
|  |  | PrivDis | -20.8010 | 7.77882 | .058 | -42.0756 | .4735 |
|  |  | PubDis | 1.5459 | 7.77882 | 1.000 | -19.7286 | 22.8205 |
|  | PrivDis | CostPun | -10.5651 | 7.23633 | .624 | -30.3559 | 9.2258 |
|  |  | NoDis | 20.8010 | 7.77882 | .058 | -.4735 | 42.0756 |
|  |  | PubDis | 22.3469^*^ | 7.47365 | .025 | 1.9070 | 42.7869 |
|  | PubDis | CostPun | -32.9120^*^ | 7.23633 | <.001 | -52.7029 | -13.1211 |
|  |  | NoDis | -1.5459 | 7.77882 | 1.000 | -22.8205 | 19.7286 |
|  |  | PrivDis | -22.3469^*^ | 7.47365 | .025 | -42.7869 | -1.9070 |
| Based on observed means.  The error term is Mean Square(Error) = 390.988. | | | | | | | |
| *. The mean difference is significant at the .05 level. | | | | | | | |

CostPun = Costly Punishment

NoDis = Nothing (i.e., No Disapproval or Punishment)

PrivDis = Private Disapproval

PubDis = Public Disapproval

## S2.3 Contributions in Early & Late Rounds, After Redefining Early & Late

To give disapproval its best chance against costly punishment (i.e., against our hypothesis that these conditions would differ), the main text classified “early” and “late” rounds based on the data where contributions increased in Public Disapproval until round 7 and then declined. To show the robustness of our results, we re-present the same analyses with three different definitions of “early” and “late” rounds: 1) S2.2.1 uses the first and last five rounds (our a priori definition of early and late), 2) S2.2.2 uses the first and last ten rounds; 3) S2.2.3 gives just the very first and last round (i.e., rounds 1 and 40). These reanalyses produce qualitatively similar results except for the minor details noted below.

### **S2.3.1. “Early” and “late” rounds as the first and last five rounds**

Table S2.3.1: Summary of means and trends of group contributions (as a percent of total endowment) with alternate definition of “early” and “late” rounds (first & last 5 rounds). The qualitative trends are the same as with the definition used in main text (first & last 7 rounds).

The omnibus test was significant, indicating that there were significant differences in both early and late rounds (Early rounds only: F_3,52_ = 6.25, p = .001, partial η^2^ = .27; Late rounds only: F_3,52_ = 10.63, p < .001, partial η^2^ = .38). There were also significant condition X round interactions in the early rounds, but this interaction was not significant in the late rounds (early rounds: F_12,208_ = 2.82, p = .028, partial η^2^ = .12; late rounds: F_12,208_ = 1.30, p = .24, partial η^2^ = .07).

Our main prediction was that contributions would be higher with Costly Punishment than with Public Disapproval, especially in the later rounds. Matching this prediction, although there was little difference between these conditions in the early rounds, there was a significant difference in the later rounds (Early rounds: Mean Difference = 5.5 ± s.e. 5.0 [-7.9, 18.9], p = .70; Later rounds: Mean Difference = 34.4 ± s.e. 7.1 [15.4, 53.3], p < .001).

Compared to our main control condition (“Nothing”), contributions were significantly higher in Costly Punishment in the late rounds but not significantly higher in the early rounds (Early rounds: Mean Difference = 10.0 ± s.e. 5.3 [-4.0, 23.9], p = .24; Later rounds: Mean Difference = 32.4 ± s.e. 7.5 [12.6, 52.1], p < .001); the early rounds results are different from the main text, possibly because in this analysis there had not yet been time for punishment to take full effect. Public Disapproval was not as effective compared to Nothing: contributions were slightly but not significantly higher in the Public Disapproval condition in the early rounds, and the two conditions were very similar in the late rounds (Early rounds: Mean Difference = 4.5 ± s.e. 5.4 [-9.9, 18.8], p = .84; Later rounds: Mean Difference = -2.0 ± s.e. 7.7 [-22.4, 18.3], p = .99).

### **S2.3.2 “Early” and “late” rounds as the first and last ten rounds**

Table S3.2: Summary of means and trends of group contributions (as a percent of total endowment) with alternate definition of “early” and “late” rounds (first & last 10 rounds). The qualitative trends are the same as with the definition used in main text (first & last 7 rounds). Early trends for Public Disapproval and Costly Punishment have started to diverge, but all other main text qualitative interpretations are similar.


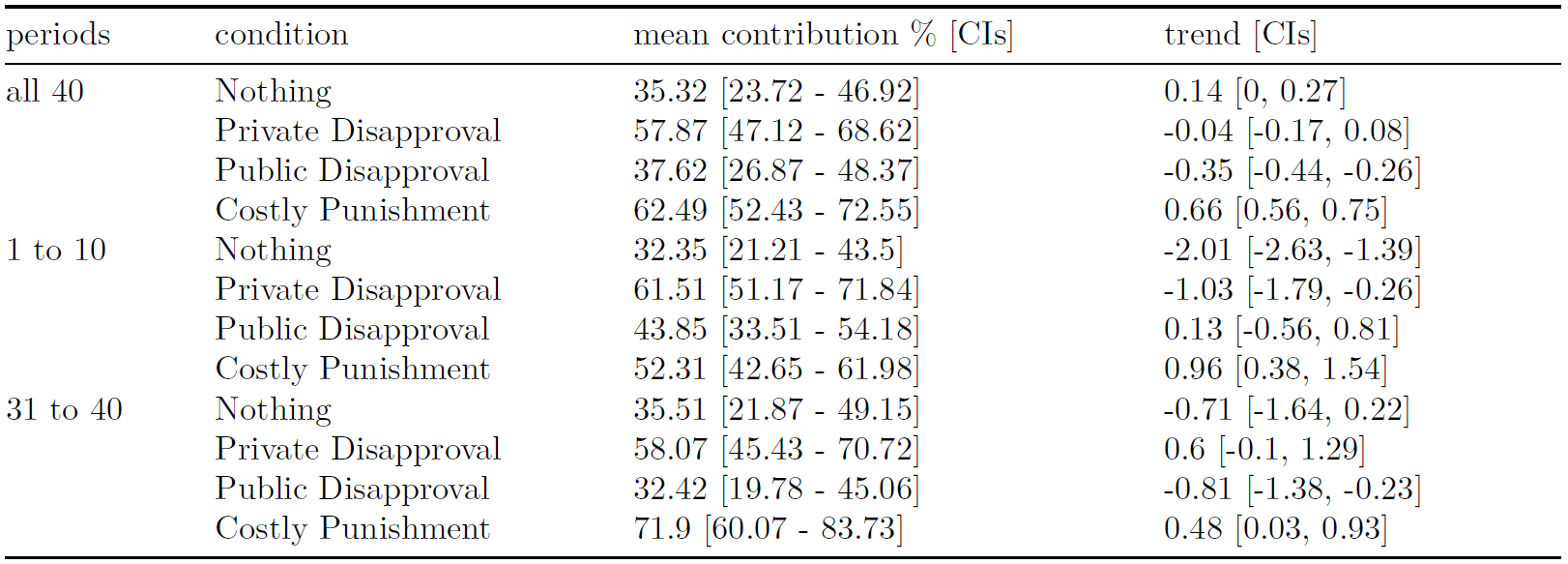


The omnibus test was significant, indicating that the there were significant differences in both the early and late rounds (Early rounds only: F_3,52_ = 5.06, p = .004, partial η^2^ = .23; Late rounds only: F_3,52_ = 8.64, p < .001, partial η^2^ = .33). There were also significant condition X round interactions in the early rounds, but in the later rounds this was marginally significant without correcting for sphericity and non-significant with a Greenhouse-Geisser correction (early rounds: F_27,468_ = 2.69, p < .001, partial η^2^ = .13; late rounds: F_27,468_ = 1.49, uncorrected p = .056, corrected p = .11, partial η^2^ = .08).

Our main prediction was that contributions would be higher with Costly Punishment than with Public Disapproval, especially in the later rounds. Matching this prediction, although there was little difference between these conditions in the early rounds, there was a significant difference in the later rounds (Early rounds: Mean Difference = 6.8 ± s.e. 5.8 [-8.7, 22.3], Tukey HSD p = .65; Later rounds: Mean Difference = 31.6 ± s.e. 7.1 [12.6, 50.5], Tukey HSD p < .001).

Compared to our main control condition (“Nothing”), contributions were higher in Costly Punishment in both the early and the late rounds (Early rounds: Mean Difference = 16.0 ± s.e. 6.1 [-0.2, 32.1], Tukey HSD *p* = .054; Later rounds: Mean Difference = 29.1 ± s.e. 7.5 [9.3, 48.9], Tukey HSD *p* = .001). Public Disapproval was not as effective compared to Nothing: contributions were non-significantly higher in the Public Disapproval condition in the early rounds, and the two conditions were very similar in the late rounds (Early rounds: Mean Difference = 9.2 ± s.e. 6.3 [-7.4, 25.8], Tukey HSD *p* = .47; Later rounds: Mean Difference = -2.5 ± s.e. 7.7 [-22.8, 17.9], Tukey HSD *p* = 0.99).

### **S2.3.3 “Early” and “late” rounds as first and last round alone (i.e., one round)**

The strictest definition of “early” and “late” rounds is the single first and single last round. This analysis will lose many of the benefits of testing punishment, given that the benefits of punishment are often achieved over time rather than instantaneously. However, it is still useful to conduct this analysis: a) to show that the Costly Punishment and Public Disapproval conditions are not different at the start of the experiment (i.e., before punishment or disapproval can occur); and b) they are different at the end.

When all four conditions are included in the analysis in first-round contributions, there is a significant difference among conditions (*F_3,52_* = 4.49, *p* < .001, partial *η^2^* = .21). However, this is almost entirely driven by the fact that the Private Disapproval had unexpectedly higher first-round contributions than the other three conditions (Private Disapproval vs. Public Disapproval: Mean Difference = 13.9 ± 4.0, [3.4, 24.5], Tukey HSD *p* = .005; Private Disapproval vs. Costly Punishment: Mean Difference = 9.8 ± s.e. 3.8, [-0.4, 20.0], Tukey HSD *p* = .065; Private Disapproval vs. Nothing: Mean Difference = 10.3 ± 4.1, [-0.7, 21.3], Tukey HSD *p* = .075); as in main text, this difference is unexplained. None of the other comparisons even approach significance and all *p* > .7 (Costly Punishment vs. Public Disapproval: Mean Difference = 4.1 ± s.e. 3.8, [-6.1, 14.4], Tukey HSD *p* = .71; Costly Punishment vs. Nothing: Mean Difference = 0.5 ± s.e. 4.0, [-10.2, 11.1], Tukey HSD *p* = 1.00; Public Disapproval vs. Nothing: Mean Difference = -3.7 ± s.e. 4.1, [-14.6, 7.3], Sidak *p* = .71). When the Private Disapproval condition is excluded, there are no significant differences between the other three conditions and the effect size is small (*F_2,39_* = 0.55, *p* =.58, partial *η^2^* = .03). This means that the Costly Punishment, Public Disapproval, and Nothing conditions all started with fairly similar contribution levels, so any later differences between them cannot be accounted for by their initial conditions.

By contrast, in the final round, there were highly significant differences among conditions (*F_3,52_* = 9.08, *p* < .001, partial *η^2^* = .34). Of particular interest, contributions were higher in Costly Punishment than in Public Disapproval and in Nothing, whereas Public Disapproval did not differ from nothing (Costly Punishment vs. Public Disapproval: Mean Difference = 36.6 ± s.e. 7.7, [16.2, 57.1], Tukey HSD *p* < .001; Costly Punishment vs. Nothing: Mean Difference = 30.3 ± s.e. 8.0, [9.0, 51.6], Tukey HSD *p* = .002; Public Disapproval vs. Nothing: Mean Difference = -6.3 ± s.e. 8.3, [-28.3, 15.6], Tukey HSD *p* = .87). Interestingly, and unexplainedly as in main text except as a Type I error, Private Disapproval had significantly higher contributions than Public Disapproval, non-significantly higher contributions than Nothing, and non-significantly lower contributions than Costly Punishment (Private Disapproval vs. Public Disapproval: Mean Difference = 22.9 ± s.e. 8.0, [1.8, 44.0], Tukey HSD *p* = .028; Private Disapproval vs. Nothing: 16.6 ± s.e. 8.3, [-5.4, 38.6], Tukey HSD *p* = .20; Private Disapproval vs. Costly Punishment: Mean Difference = -13.8 ± s.e. 7.7, [-34.1, 6.7], Tukey HSD *p* = .29).

## S2.4 Graphs of Individual-Level Contributions Data

In case this is of interest to any readers, we present here the public goods contributions in each group as an individual panel, with each individual within a group as an individual line coloured blue, red, yellow, and pink (in no particular order). An informal visual inspection suggests that there are some general trends across conditions. For example, contributions are visibly higher in many sessions of Costly Punishment than in Public Disapproval, as discussed in the main text.

However, arguably more striking is that there is much variation between sessions, between individuals within a session, and especially within individuals. For example, in the sessions with Costly Punishment, some groups coordinated relatively quickly on high contributions, whereas others bounced around and had intermediate contributions, and some coordinated on relatively low contributions. In other cases, there were consistent individual differences within a session, where some participants consistently contributed high amounts and others consistently contributed low amounts (e.g., compare the blue vs. yellow lines in the final session of Nothing, 13031913392, at the bottom right).

Most notably, many individuals fluctuated wildly in their contributions, sometimes even alternating between contributing near the maximum and near the minimum. For example, in session 12112913371 of the Nothing Condition (middle row, leftmost panel), both the red and blue lines both have multiple rounds of maximum contributions followed by minimum contributions. Nor is this session unique – there are many sessions with participants doing something similar. We did not expect such variation within individuals, as most researchers discuss cooperation as if it were a stable individual trait, albeit subject to conditional cooperation (i.e., cooperating only if others do likewise). We are not sure what causes such fluctuations in contributions, but we can speculate on some potential reasons that are not mutually exclusive. For example, participants might fluctuate because they are confused and are testing out possible responses (e.g., payoff-based learning, Burton-Chellew & West 2021), because they are conditional cooperators sending strong messages in response to other participants’ behaviour, or simply because they are bored. We can also speculate on their effect, in that large individual-level fluctuations may help push a group from one point they are coordinating on to a different coordination point (e.g., a cooperative “shock” to help a group recover from low contributions).

A full analysis of individual-level contributions is far beyond the scope of this paper – our focus is on the long-term effects costly punishment vs. cheap disapproval. We present them out of completeness, and in case this inspires future researchers to examine these wide fluctuations in contributions in future work, what causes these wide fluctuations, and what impact they have on group cooperation.

**

Figure S2.4.1: Individual-level contributions in each session of the Nothing condition.

Figure S2.4.2: Individual-level contributions in each session of the Costly Punishment condition.


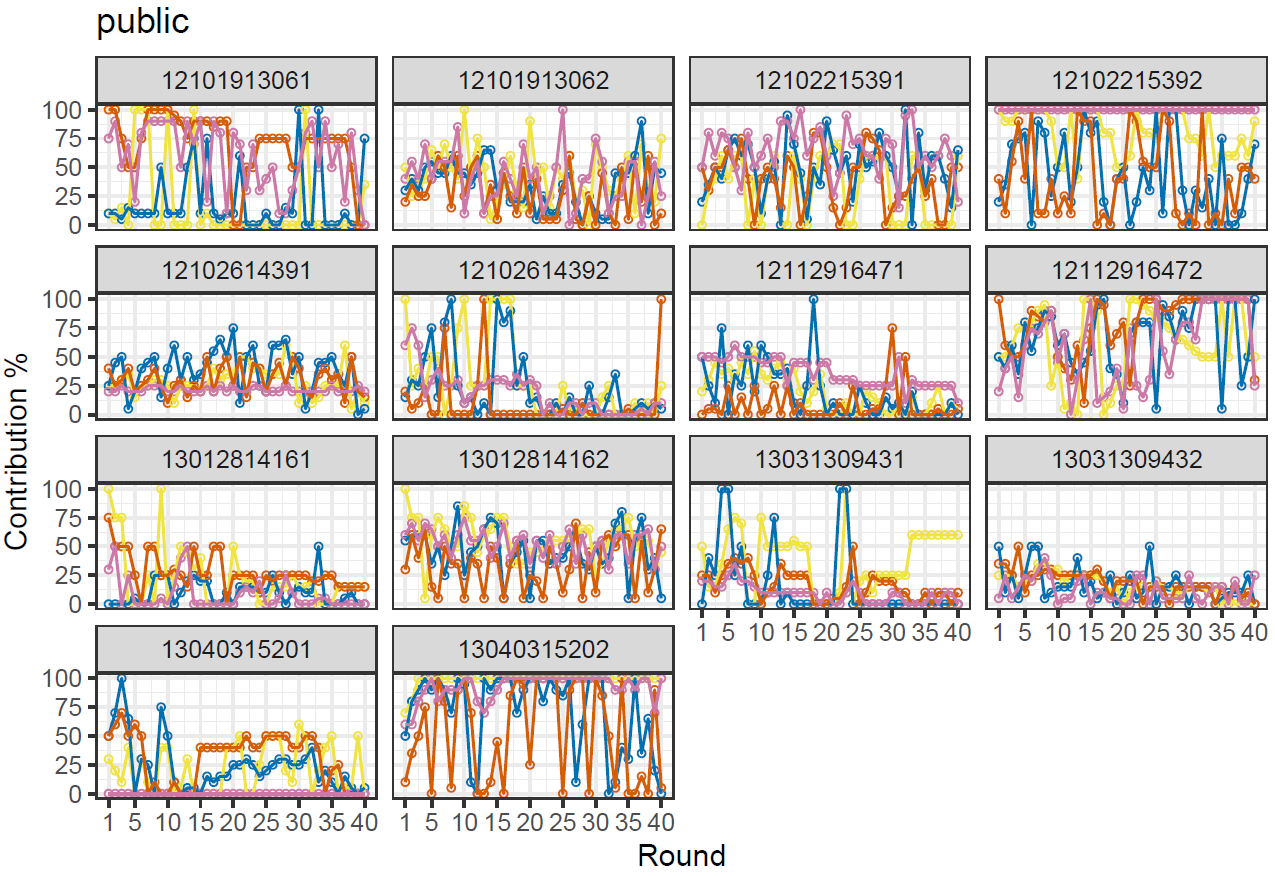


Figure S2.4.3: Individual-level contributions in each session of the Public Disapproval condition.

Figure S2.4.4: Individual-level contributions in each session of the Private Disapproval condition

## S2.5 Contributions in the 1^st^ vs. 2^nd^ Chronological Half of the Sessions

The main text reported that contributions were surprisingly high in the Private Disapproval condition. However, after seeing the individual-level contributions in Figure S2.4.4, we wondered if this were just a statistical fluke occurring in the first sessions that were conducted in this condition. To test this, we split each condition chronologically in half, i.e., we separated the first several sessions that were conducted in each condition from the second several sessions that were conducted. Figure S2.5 presents the data from this post hoc split:


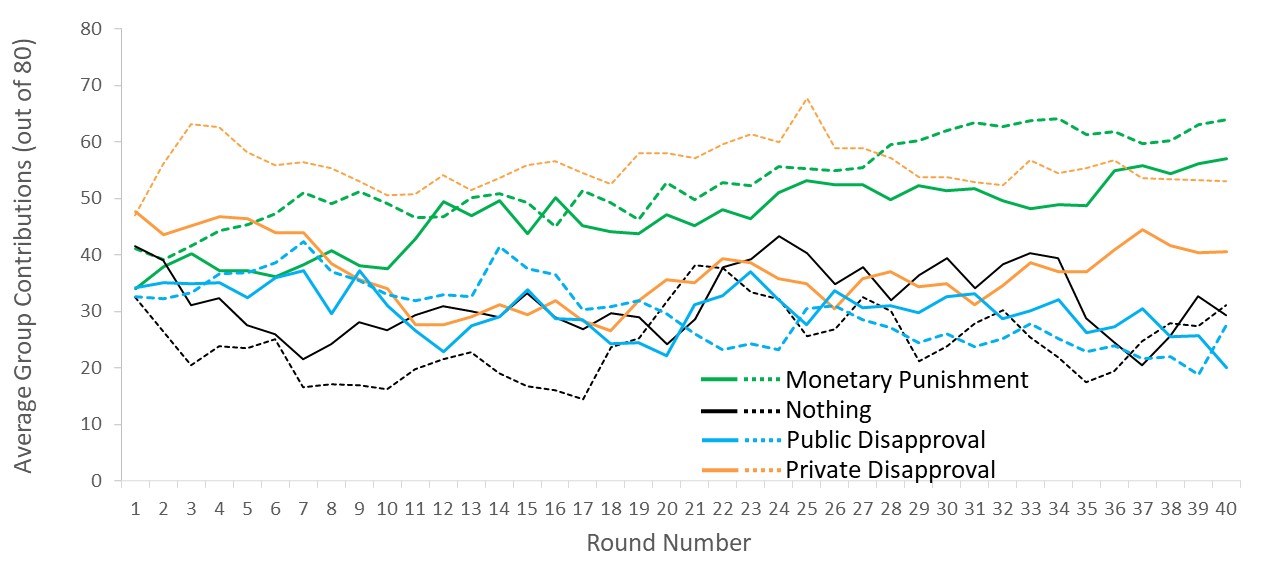


Figure S2.5: Average contributions across 40 rounds of the public goods game with Monetary Punishment (green lines), Public Disapproval (blue lines), Private Disapproval (orange lines), and Nothing (black lines). Within each condition, we present separately the sessions that were chronologically conducted first (dashed lines) or second (solid lines). We omitted SEM to make it easier to see the patterns – a graph with SEM is available in section S2.5.1.

As can be seen in Figure S2.5, in all conditions except Private Disapproval, the first half of the groups had similar contribution levels to the second half. However, in Private Disapproval, the two halves differed: the first half of the groups had the surprisingly high contributions (dashed orange line), whereas the second half of the groups (solid orange line) had lower contributions that were more similar to Nothing. Table S2.5 shows the Tukey HSD post hoc comparisons from a GLM with the Private Disapproval condition split chronologically: despite the low power, the first groups of Private Disapproval have significantly higher contributions than Public Disapproval and Nothing (both *p*s < .01), whereas the later groups of Private Disapproval are much closer to Public Disapproval and Nothing and are statistically similar (all *p*s > .80). If we split *all* conditions chronologically, then the results are similar, but the much lower power makes the significant differences only marginally significant; in all other conditions the two chronological halves do not even approach a significant difference (all *p*s > .90, Table S2.5.1). This post hoc analysis supports the suggestion that the first several groups in Private Disapproval were a statistical fluke that was not replicated in the next set of groups.

Another finding is worth noting from a visual inspection of Figure S2.5. In both halves of the Nothing condition (black lines), contributions dropped at first but later spontaneously recovered. This replication suggests that this spontaneous recovery is *not* just a statistical fluke from some groups, given that it occurred independently in both halves. Furthermore, this spontaneous recovery seemed to also occur in the second half of the Private Disapproval groups (solid orange line), which a) makes those groups again more similar to the Nothing condition; and b) further suggests that spontaneous recovery might occur more often if experimenters conducted experiments that lasted long enough to find it (>20 rounds).

*Table S2.5: Tukey post-hoc tests comparing average contributions (across 40 rounds) in the first and second halves of the groups in the Private Disapproval Condition.*

| **Multiple Comparisons** | | | | | | |
| --- | --- | --- | --- | --- | --- | --- |
| Measure: MEASURE_1 | | | | | | |
| Tukey HSD | | | | | | |
| (I) splitPriv | (J) splitPriv | Mean Difference (I-J) | Std. Error | Sig. | 95% Confidence Interval | |
|  |  |  |  |  | Lower Bound | Upper Bound |
| CostPun | NoDis | 21.7359^*^ | 6.11860 | .007 | 4.4340 | 39.0379 |
|  | PrivDis1 | -5.8864 | 7.26070 | .926 | -26.4179 | 14.6451 |
|  | PrivDis2 | 13.2815 | 7.26070 | .368 | -7.2500 | 33.8130 |
|  | PubDis | 19.8940^*^ | 5.86355 | .011 | 3.3133 | 36.4747 |
| NoDis | CostPun | -21.7359^*^ | 6.11860 | .007 | -39.0379 | -4.4340 |
|  | PrivDis1 | -27.6223^*^ | 7.62010 | .006 | -49.1701 | -6.0745 |
|  | PrivDis2 | -8.4545 | 7.62010 | .801 | -30.0023 | 13.0934 |
|  | PubDis | -1.8420 | 6.30312 | .998 | -19.6657 | 15.9817 |
| PrivDis1 | CostPun | 5.8864 | 7.26070 | .926 | -14.6451 | 26.4179 |
|  | NoDis | 27.6223^*^ | 7.62010 | .006 | 6.0745 | 49.1701 |
|  | PrivDis2 | 19.1679 | 8.56426 | .182 | -5.0498 | 43.3855 |
|  | PubDis | 25.7804^*^ | 7.41686 | .009 | 4.8073 | 46.7535 |
| PrivDis2 | CostPun | -13.2815 | 7.26070 | .368 | -33.8130 | 7.2500 |
|  | NoDis | 8.4545 | 7.62010 | .801 | -13.0934 | 30.0023 |
|  | PrivDis1 | -19.1679 | 8.56426 | .182 | -43.3855 | 5.0498 |
|  | PubDis | 6.6125 | 7.41686 | .899 | -14.3606 | 27.5856 |
| PubDis | CostPun | -19.8940^*^ | 5.86355 | .011 | -36.4747 | -3.3133 |
|  | NoDis | 1.8420 | 6.30312 | .998 | -15.9817 | 19.6657 |
|  | PrivDis1 | -25.7804^*^ | 7.41686 | .009 | -46.7535 | -4.8073 |
|  | PrivDis2 | -6.6125 | 7.41686 | .899 | -27.5856 | 14.3606 |
| Based on observed means.  The error term is Mean Square(Error) = 256.713. | | | | | | |
| *. The mean difference is significant at the .05 level. | | | | | | |

CostPun = Costly Punishment

NoDis = Nothing (i.e., No Disapproval or Punishment)

PrivDis1 & PrivDis2 = Private Disapproval, first & second halves of the sessions

PubDis = Public Disapproval

### S2.5.1 Supplement About the First & Second Chronological Halves of the Sessions

In the above section, we presented the results of our post hoc analysis as accessibly as possible. Here we present the same results, but with more detail on two points. First, we re-present Figure S2.5 of the first and second chronological halves, but with error bars. We present this will all conditions (upper panel), but we also split it into two graphs to make it easier to compare the two halves of each condition (middle & lower panels). Note that the Private Disapproval condition is the only condition where the error bars regularly fail to overlap between the first and second chronological halves. In fact, there ten times as many non-overlaps in the Private Disapproval condition (20 of the 40 rounds) than in all three other conditions combined (2 of the 40 rounds in the Costly Punishment Condition). This visual impression suggests that the first half of the Private Disapproval sessions produced a result that did not replicate in the second half, whereas all other conditions replicated the same basic pattern in both halves.

Second, we support this visual impression with statistics in Table 2.5.1. Despite the low statistical power, the first half of the Private Disapproval sessions are marginally significantly different from both halves of Public Disapproval (*p* = .080 & .087) and from one of the No Punishment halves (*p* = .025). By contrast, the second half of the Private Disapproval sessions is statistically indistinguishable from these other conditions (*p*s = .99, .99, .88, and .99). Furthermore, in all other conditions, the two chronological halves are statistically indistinguishable from each other (all *p* > .99). Altogether, this supports the idea that the surprisingly high contributions in Private Disapproval were a statistical fluke in the first several sessions that was not replicated in the later sessions.


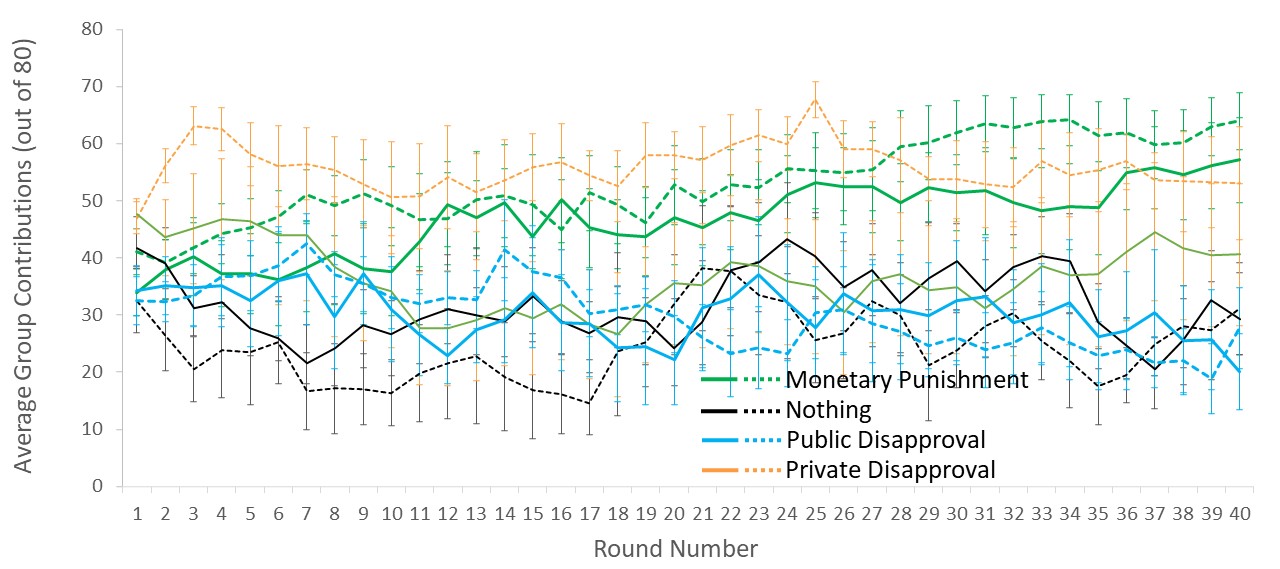


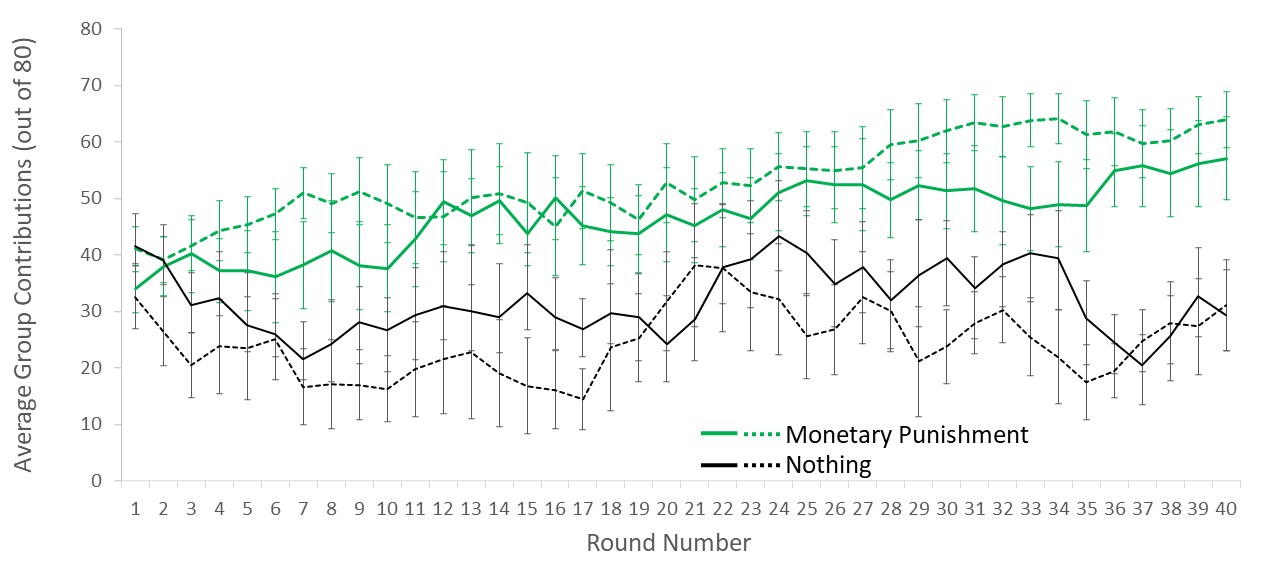


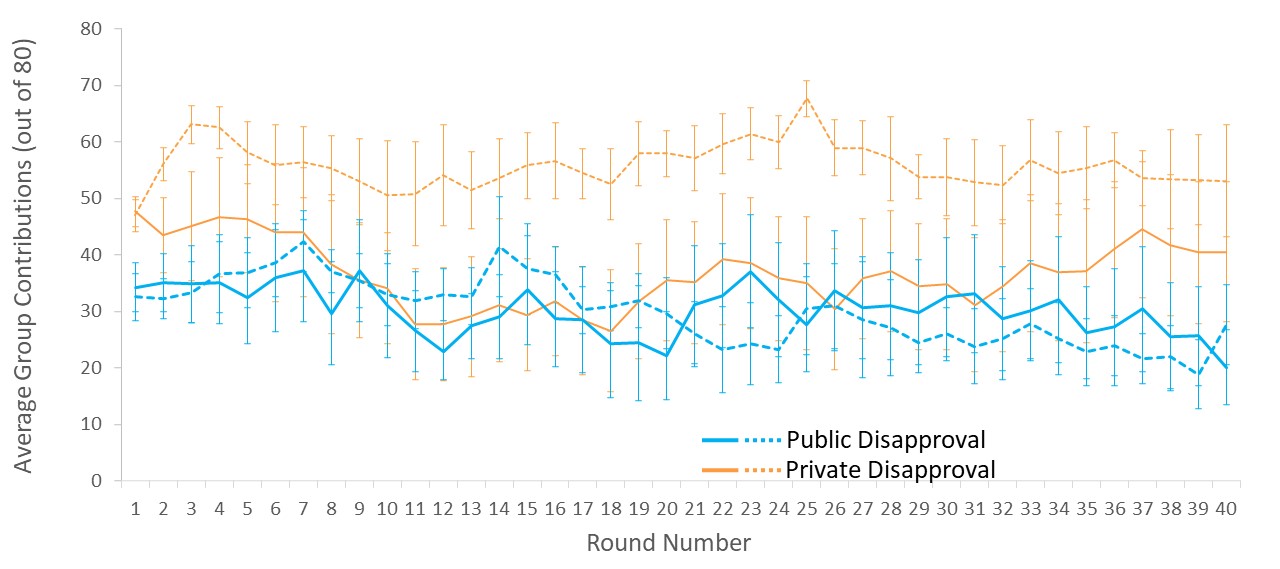


Figure S2.5: Average contributions across 40 rounds of the public goods game with: Monetary Punishment (green lines), Public Disapproval (blue lines), Private Disapproval (orange lines), and Nothing (black lines). Within each condition, we present separately the sessions that were chronologically conducted first (dashed lines) or second (solid lines).

*Table S2.5.1: Tukey post-hoc tests comparing average contributions (across 40 rounds) in the first and second halves of the groups in each condition (which has greatly reduced statistical power).*

| **Multiple Comparisons** | | | | | | |
| --- | --- | --- | --- | --- | --- | --- |
| Measure: MEASURE_1 | | | | | | |
| Tukey HSD | | | | | | |
| (I) splitconditions | (J) splitconditions | Mean Difference (I-J) | Std. Error | Sig. | 95% Confidence Interval | |
|  |  |  |  |  | Lower Bound | Upper Bound |
| CostPun1 | CostPun2 | -6.5344 | 8.15420 | .992 | -32.3692 | 19.3005 |
|  | NoPun1 | 22.0625 | 8.80755 | .218 | -5.8423 | 49.9673 |
|  | NoPun2 | 14.8750 | 8.80755 | .694 | -13.0298 | 42.7798 |
|  | PrivDis1 | -9.1536 | 8.44040 | .957 | -35.8952 | 17.5880 |
|  | PrivDis2 | 10.0143 | 8.44040 | .932 | -16.7273 | 36.7559 |
|  | PubDis1 | 16.7714 | 8.44040 | .501 | -9.9702 | 43.5130 |
|  | PubDis2 | 16.4821 | 8.44040 | .523 | -10.2595 | 43.2237 |
| CostPun2 | CostPun1 | 6.5344 | 8.15420 | .992 | -19.3005 | 32.3692 |
|  | NoPun1 | 28.5969^*^ | 8.80755 | .041 | .6921 | 56.5017 |
|  | NoPun2 | 21.4094 | 8.80755 | .250 | -6.4954 | 49.3142 |
|  | PrivDis1 | -2.6192 | 8.44040 | 1.000 | -29.3608 | 24.1224 |
|  | PrivDis2 | 16.5487 | 8.44040 | .518 | -10.1929 | 43.2903 |
|  | PubDis1 | 23.3058 | 8.44040 | .130 | -3.4358 | 50.0474 |
|  | PubDis2 | 23.0165 | 8.44040 | .139 | -3.7251 | 49.7581 |
| NoPun1 | CostPun1 | -22.0625 | 8.80755 | .218 | -49.9673 | 5.8423 |
|  | CostPun2 | -28.5969^*^ | 8.80755 | .041 | -56.5017 | -.6921 |
|  | NoPun2 | -7.1875 | 9.41566 | .994 | -37.0190 | 22.6440 |
|  | PrivDis1 | -31.2161^*^ | 9.07316 | .025 | -59.9624 | -2.4697 |
|  | PrivDis2 | -12.0482 | 9.07316 | .884 | -40.7946 | 16.6981 |
|  | PubDis1 | -5.2911 | 9.07316 | .999 | -34.0374 | 23.4553 |
|  | PubDis2 | -5.5804 | 9.07316 | .999 | -34.3267 | 23.1660 |
| NoPun2 | CostPun1 | -14.8750 | 8.80755 | .694 | -42.7798 | 13.0298 |
|  | CostPun2 | -21.4094 | 8.80755 | .250 | -49.3142 | 6.4954 |
|  | NoPun1 | 7.1875 | 9.41566 | .994 | -22.6440 | 37.0190 |
|  | PrivDis1 | -24.0286 | 9.07316 | .164 | -52.7749 | 4.7178 |
|  | PrivDis2 | -4.8607 | 9.07316 | .999 | -33.6071 | 23.8856 |
|  | PubDis1 | 1.8964 | 9.07316 | 1.000 | -26.8499 | 30.6428 |
|  | PubDis2 | 1.6071 | 9.07316 | 1.000 | -27.1392 | 30.3535 |
| PrivDis1 | CostPun1 | 9.1536 | 8.44040 | .957 | -17.5880 | 35.8952 |
|  | CostPun2 | 2.6192 | 8.44040 | 1.000 | -24.1224 | 29.3608 |
|  | NoPun1 | 31.2161^*^ | 9.07316 | .025 | 2.4697 | 59.9624 |
|  | NoPun2 | 24.0286 | 9.07316 | .164 | -4.7178 | 52.7749 |
|  | PrivDis2 | 19.1679 | 8.71721 | .371 | -8.4508 | 46.7865 |
|  | PubDis1 | 25.9250 | 8.71721 | .080 | -1.6936 | 53.5436 |
|  | PubDis2 | 25.6357 | 8.71721 | .087 | -1.9829 | 53.2543 |
| PrivDis2 | CostPun1 | -10.0143 | 8.44040 | .932 | -36.7559 | 16.7273 |
|  | CostPun2 | -16.5487 | 8.44040 | .518 | -43.2903 | 10.1929 |
|  | NoPun1 | 12.0482 | 9.07316 | .884 | -16.6981 | 40.7946 |
|  | NoPun2 | 4.8607 | 9.07316 | .999 | -23.8856 | 33.6071 |
|  | PrivDis1 | -19.1679 | 8.71721 | .371 | -46.7865 | 8.4508 |
|  | PubDis1 | 6.7571 | 8.71721 | .994 | -20.8615 | 34.3758 |
|  | PubDis2 | 6.4679 | 8.71721 | .995 | -21.1508 | 34.0865 |
| PubDis1 | CostPun1 | -16.7714 | 8.44040 | .501 | -43.5130 | 9.9702 |
|  | CostPun2 | -23.3058 | 8.44040 | .130 | -50.0474 | 3.4358 |
|  | NoPun1 | 5.2911 | 9.07316 | .999 | -23.4553 | 34.0374 |
|  | NoPun2 | -1.8964 | 9.07316 | 1.000 | -30.6428 | 26.8499 |
|  | PrivDis1 | -25.9250 | 8.71721 | .080 | -53.5436 | 1.6936 |
|  | PrivDis2 | -6.7571 | 8.71721 | .994 | -34.3758 | 20.8615 |
|  | PubDis2 | -.2893 | 8.71721 | 1.000 | -27.9079 | 27.3293 |
| PubDis2 | CostPun1 | -16.4821 | 8.44040 | .523 | -43.2237 | 10.2595 |
|  | CostPun2 | -23.0165 | 8.44040 | .139 | -49.7581 | 3.7251 |
|  | NoPun1 | 5.5804 | 9.07316 | .999 | -23.1660 | 34.3267 |
|  | NoPun2 | -1.6071 | 9.07316 | 1.000 | -30.3535 | 27.1392 |
|  | PrivDis1 | -25.6357 | 8.71721 | .087 | -53.2543 | 1.9829 |
|  | PrivDis2 | -6.4679 | 8.71721 | .995 | -34.0865 | 21.1508 |
|  | PubDis1 | .2893 | 8.71721 | 1.000 | -27.3293 | 27.9079 |
| Based on observed means.  The error term is Mean Square(Error) = 265.964. | | | | | | |
| *. The mean difference is significant at the .05 level. | | | | | | |

CostPun1 & CostPun2 = Costly Punishment, first & second halves of the sessions

NoPun1 & NoPun2 = Nothing (i.e., No Disapproval or Punishment)

PrivDis1 & PrivDis2 = Private Disapproval, first & second halves of the sessions

PubDis1 & PubDis2 = Public Disapproval, first & second halves of the sessions

# S3 Supplementary Information on Punishment

## S3.1 Means and Trends for Disapproval and Punishment

Table S3.1: Summary of means and trends for disapproval and punishment

## S3.2 Post Hoc Tests of Between-Condition Differences in Punishment/Disapproval

The main text compares the levels of punishment and disapproval in the three conditions that have them (Costly Punishment, Public Disapproval, Private Disapproval). Here we present the full tables of post hoc comparisons of earnings per round in all conditions using Tukey HSD, Scheffe, and Sidak tests from our Repeated Measures GLM in SPSS with the three between-subjects conditions. The results are similar for all three post hoc tests.

### **S3.2.1 Post Hoc Tests of Punishment/Disapproval in All 40 Rounds**

*Table S3.2.1 Post hoc tests of punishment/disapproval in all 40 rounds*

| **Multiple Comparisons** | | | | | | | |
| --- | --- | --- | --- | --- | --- | --- | --- |
| Measure: MEASURE_1 | | | | | | | |
|  | (I) condition | (J) condition | Mean Difference (I-J) | Std. Error | Sig. | 95% Confidence Interval | |
|  |  |  |  |  |  | Lower Bound | Upper Bound |
| Tukey HSD | CostPun | PrivDis | -36.9368^*^ | 6.25963 | <.001 | -52.1581 | -21.7156 |
|  |  | PubDis | -51.9672^*^ | 6.25963 | <.001 | -67.1884 | -36.7459 |
|  | PrivDis | CostPun | 36.9368^*^ | 6.25963 | <.001 | 21.7156 | 52.1581 |
|  |  | PubDis | -15.0304 | 6.46492 | .063 | -30.7508 | .6901 |
|  | PubDis | CostPun | 51.9672^*^ | 6.25963 | <.001 | 36.7459 | 67.1884 |
|  |  | PrivDis | 15.0304 | 6.46492 | .063 | -.6901 | 30.7508 |
| Scheffe | CostPun | PrivDis | -36.9368^*^ | 6.25963 | <.001 | -52.8360 | -21.0376 |
|  |  | PubDis | -51.9672^*^ | 6.25963 | <.001 | -67.8664 | -36.0680 |
|  | PrivDis | CostPun | 36.9368^*^ | 6.25963 | <.001 | 21.0376 | 52.8360 |
|  |  | PubDis | -15.0304 | 6.46492 | .079 | -31.4510 | 1.3903 |
|  | PubDis | CostPun | 51.9672^*^ | 6.25963 | <.001 | 36.0680 | 67.8664 |
|  |  | PrivDis | 15.0304 | 6.46492 | .079 | -1.3903 | 31.4510 |
| Sidak | CostPun | PrivDis | -36.9368^*^ | 6.25963 | <.001 | -52.5184 | -21.3552 |
|  |  | PubDis | -51.9672^*^ | 6.25963 | <.001 | -67.5488 | -36.3856 |
|  | PrivDis | CostPun | 36.9368^*^ | 6.25963 | <.001 | 21.3552 | 52.5184 |
|  |  | PubDis | -15.0304 | 6.46492 | .073 | -31.1230 | 1.0623 |
|  | PubDis | CostPun | 51.9672^*^ | 6.25963 | <.001 | 36.3856 | 67.5488 |
|  |  | PrivDis | 15.0304 | 6.46492 | .073 | -1.0623 | 31.1230 |
| Based on observed means.  The error term is Mean Square(Error) = 292.567. | | | | | | | |
| *. The mean difference is significant at the .05 level. | | | | | | | |

CostPun = Costly Punishment

PrivDis = Private Disapproval

PubDis = Public Disapproval

### **3.2.2 Post Hoc Tests of Punishment/Disapproval in Early Rounds (i.e., Rounds 1-7)**

*Table S3.2.2 Post hoc tests of punishment/disapproval in early rounds (i.e., Rounds 1-7)*

| **Multiple Comparisons** | | | | | | | |
| --- | --- | --- | --- | --- | --- | --- | --- |
| Measure: MEASURE_1 | | | | | | | |
|  | (I) condition | (J) condition | Mean Difference (I-J) | Std. Error | Sig. | 95% Confidence Interval | |
|  |  |  |  |  |  | Lower Bound | Upper Bound |
| Tukey HSD | CostPun | PrivDis | -33.3189^*^ | 5.96814 | <.001 | -47.8313 | -18.8064 |
|  |  | PubDis | -40.3189^*^ | 5.96814 | <.001 | -54.8313 | -25.8064 |
|  | PrivDis | CostPun | 33.3189^*^ | 5.96814 | <.001 | 18.8064 | 47.8313 |
|  |  | PubDis | -7.0000 | 6.16387 | .498 | -21.9884 | 7.9884 |
|  | PubDis | CostPun | 40.3189^*^ | 5.96814 | <.001 | 25.8064 | 54.8313 |
|  |  | PrivDis | 7.0000 | 6.16387 | .498 | -7.9884 | 21.9884 |
| Scheffe | CostPun | PrivDis | -33.3189^*^ | 5.96814 | <.001 | -48.4777 | -18.1601 |
|  |  | PubDis | -40.3189^*^ | 5.96814 | <.001 | -55.4777 | -25.1601 |
|  | PrivDis | CostPun | 33.3189^*^ | 5.96814 | <.001 | 18.1601 | 48.4777 |
|  |  | PubDis | -7.0000 | 6.16387 | .530 | -22.6560 | 8.6560 |
|  | PubDis | CostPun | 40.3189^*^ | 5.96814 | <.001 | 25.1601 | 55.4777 |
|  |  | PrivDis | 7.0000 | 6.16387 | .530 | -8.6560 | 22.6560 |
| Sidak | CostPun | PrivDis | -33.3189^*^ | 5.96814 | <.001 | -48.1749 | -18.4629 |
|  |  | PubDis | -40.3189^*^ | 5.96814 | <.001 | -55.1749 | -25.4629 |
|  | PrivDis | CostPun | 33.3189^*^ | 5.96814 | <.001 | 18.4629 | 48.1749 |
|  |  | PubDis | -7.0000 | 6.16387 | .599 | -22.3432 | 8.3432 |
|  | PubDis | CostPun | 40.3189^*^ | 5.96814 | <.001 | 25.4629 | 55.1749 |
|  |  | PrivDis | 7.0000 | 6.16387 | .599 | -8.3432 | 22.3432 |
| Based on observed means.  The error term is Mean Square(Error) = 265.953. | | | | | | | |
| *. The mean difference is significant at the .05 level. | | | | | | | |

CostPun = Costly Punishment

PrivDis = Private Disapproval

PubDis = Public Disapproval

### **S3.2.3 Post Hoc Tests of Punishment/Disapproval in Late Rounds (i.e., Rounds 34-40)**

*Table S3.2.3 Post hoc tests of punishment/disapproval in late rounds (i.e., Rounds 34-40)*

| **Multiple Comparisons** | | | | | | | |
| --- | --- | --- | --- | --- | --- | --- | --- |
| Measure: MEASURE_1 | | | | | | | |
|  | (I) condition | (J) condition | Mean Difference (I-J) | Std. Error | Sig. | 95% Confidence Interval | |
|  |  |  |  |  |  | Lower Bound | Upper Bound |
| Tukey HSD | CostPun | PrivDis | -36.9133^*^ | 7.52851 | <.001 | -55.2200 | -18.6066 |
|  |  | PubDis | -61.0357^*^ | 7.52851 | <.001 | -79.3424 | -42.7290 |
|  | PrivDis | CostPun | 36.9133^*^ | 7.52851 | <.001 | 18.6066 | 55.2200 |
|  |  | PubDis | -24.1224^*^ | 7.77541 | .010 | -43.0295 | -5.2154 |
|  | PubDis | CostPun | 61.0357^*^ | 7.52851 | <.001 | 42.7290 | 79.3424 |
|  |  | PrivDis | 24.1224^*^ | 7.77541 | .010 | 5.2154 | 43.0295 |
| Scheffe | CostPun | PrivDis | -36.9133^*^ | 7.52851 | <.001 | -56.0353 | -17.7912 |
|  |  | PubDis | -61.0357^*^ | 7.52851 | <.001 | -80.1578 | -41.9136 |
|  | PrivDis | CostPun | 36.9133^*^ | 7.52851 | <.001 | 17.7912 | 56.0353 |
|  |  | PubDis | -24.1224^*^ | 7.77541 | .013 | -43.8716 | -4.3733 |
|  | PubDis | CostPun | 61.0357^*^ | 7.52851 | <.001 | 41.9136 | 80.1578 |
|  |  | PrivDis | 24.1224^*^ | 7.77541 | .013 | 4.3733 | 43.8716 |
| Sidak | CostPun | PrivDis | -36.9133^*^ | 7.52851 | <.001 | -55.6534 | -18.1731 |
|  |  | PubDis | -61.0357^*^ | 7.52851 | <.001 | -79.7758 | -42.2956 |
|  | PrivDis | CostPun | 36.9133^*^ | 7.52851 | <.001 | 18.1731 | 55.6534 |
|  |  | PubDis | -24.1224^*^ | 7.77541 | .010 | -43.4772 | -4.7677 |
|  | PubDis | CostPun | 61.0357^*^ | 7.52851 | <.001 | 42.2956 | 79.7758 |
|  |  | PrivDis | 24.1224^*^ | 7.77541 | .010 | 4.7677 | 43.4772 |
| Based on observed means.  The error term is Mean Square(Error) = 423.199. | | | | | | | |
| *. The mean difference is significant at the .05 level. | | | | | | | |

CostPun = Costly Punishment

PrivDis = Private Disapproval

PubDis = Public Disapproval

## S3.3 Closer Visualizations of Costly Punishment at the Session Level

Figure S1 presented punishment and disapproval over all 40 rounds and the density plot for disapproval and punishment. However, because there was more disapproval than costly punishment, our use of a common scale makes it harder to see the differences in the Costly Punishment condition. In this section, we present some of the same data as in Figure S1, but on scales that makes it easier to see the patterns in Costly Punishment. In particular, this zoom-in shows that most groups had little to no costly punishment, especially in the later rounds.

Figure S3.3.1: A closer analysis of punishment in the Costly Punishment condition over time - these data were presented as part of Figure S1 but on a different scale. The colored solid line and shaded 95% confidence intervals summarize period mean trends using LOESS smoothing; grey dashed lines summarize group mean trends with LOESS smoothing. As can be seen, most groups had little or no punishment, especially later in the experiment.

Figure S3.3.2: Density plot of punishment and disapproval. These data were presented in truncated form in Figure S1 (second row, right panel); here they are displayed without truncation.

## S3.4 Punishment Received in Early and Late Rounds

Here we present a similar graph to Figure 3 in the main text, except broken down by early and late rounds. The patterns are generally similar in early and late, with minor variations. We observe the “Hypocrisy Valley” at both time periods, i.e., disapproval and punishment are much lower towards others who contribute nearly the same as oneself compared with the rest of the curve.

Figure S3.4: Player-level data on negative reactions to other players as a function of how much more or less that other person contributed relative to oneself, presented by early rounds (1-7) and late rounds (34-40). Each point represents one of the three individual punishment or disapproval decisions each player makes in each round (i.e., one for each other group member), with jitter. Curves are smoothed using Generalized Additive Models.

## S3.5 Effects of Receiving Punishment

We were primarily interested in how the *presence* of costly punishment vs. disapproval affects contributions. A related question is how participants change their behaviour in the round after personally *receiving* costly punishment vs. disapproval. Although this latter question is not our primary interest, some readers will be interested in this question, so we present graphs showing how participants change their contributions after receiving different amounts of costly punishment and disapproval. We differentiate between punishment or disapproval targeting lower-than-average contributors (“prosocial punishment”) vs. higher-than-average contributors (“antisocial punishment”), because these are typically described as separate phenomena with different goals (e.g., Herrmann et al., 2008; Pleasant & Barclay, 2018).

Figure S3.5: Player contribution change as a function of the previous period’s punishment or disapproval from other players. Data are pooled across all rounds and fit separately for above- and below-average contributors, and the curves were smoothed using Generalized Additive Models. Note that participants in the Private Disapproval condition do not actually receive the disapproval from others, so the disapproval cannot directly change anyone’s behaviour.

Both pro- and antisocial costly punishment are proportionately associated with subsequent changes in contribution: higher punishment of low contributors is associated with larger subsequent contribution increases, and higher punishment of high contributors is associated with larger subsequent contribution decreases. Disapproval of low contributors is also proportionately associated with their increased subsequent contributions, including in the Private Disapproval condition where no direct causal effect is possible because the disapproval was not conveyed to participants. Raihani and Bshary (2019) argue that low contributors see others’ high contributions and subsequently respond by contributing more themselves, but that receiving punishment has little effect beyond the effects of seeing others’ high contributions; the results of our Private Disapproval condition are consistent with that perspective. In fact, the relationship between disapproval and subsequent contributions is shallower in the Public Disapproval condition than the Private Disapproval condition (no overlap of confidence intervals in this Figure), suggesting that if anything, actually receiving the disapproval partially suppresses the increases in contributions (see also: Barr, A. (2001) Social dilemmas and shame-based sanctions: Experimental results from rural Zimbabwe. Working Paper WPS/2001.11, Centre for the Study of African Economics, University of Oxford, U.K.).

## S3.6 Graphs of Individual-Level Punishment/Dissatisfaction Received

In case this is of interest to any readers, we present here the punishment and disapproval received by each individual within a group. Each panel represents one group, and each coloured line represents an individual participant (the colours are in no particular order). The maximum possible punishment or disapproval in any one round is 30 (i.e., 10 from each other group member).

An informal visual analysis supports the analyses in the main text: participants assigned much less costly punishment than disapproval, and this is visually obvious even despite the smaller range of the y-axis of the graph for the Costly Punishment condition. In the Costly Punishment condition (S.3.4a), most groups converged fairly quickly on all group members receiving low punishment, with occasional blips.

By contrast, there was little convergence in the Public Disapproval or Private Disapproval conditions (S.3.4b and S.3.4c, respectively). In these conditions, there was both between- and within-individual variation: some participants consistently received more or less disapproval than other participants, whereas other participants fluctuated between near-maximum and near-minimum disapproval. These fluctuations are likely due to the large fluctuations in contributions, discussed in above in section S2.3. It is beyond the scope of this paper to provide a full analysis of this variation – our focus is on the long-term effects of the presence of monetary punishment vs. disapproval. Instead, we just present them out of completeness, and to inspire future researchers to examine this individual variation in their studies.

Figure S3.6.1: Punishment received by individuals in each group in each round of the Costly Punishment condition.

Figure S3.6.2: Disapproval received by individuals in each group in each round of the Public Disapproval condition.

Figure S3.6.3: Disapproval targeted at each individual in each group in each round of Private Disapproval. Reminder: disapproval was not conveyed to participants in this condition.

# S4 Supplementary Information on Profits

## S4.1 Table of Means and Trends of Profits

Table S2: Summary of means and trends of profits

## S4.2 Session-Level Graphs of Profits in Early vs. Late Rounds


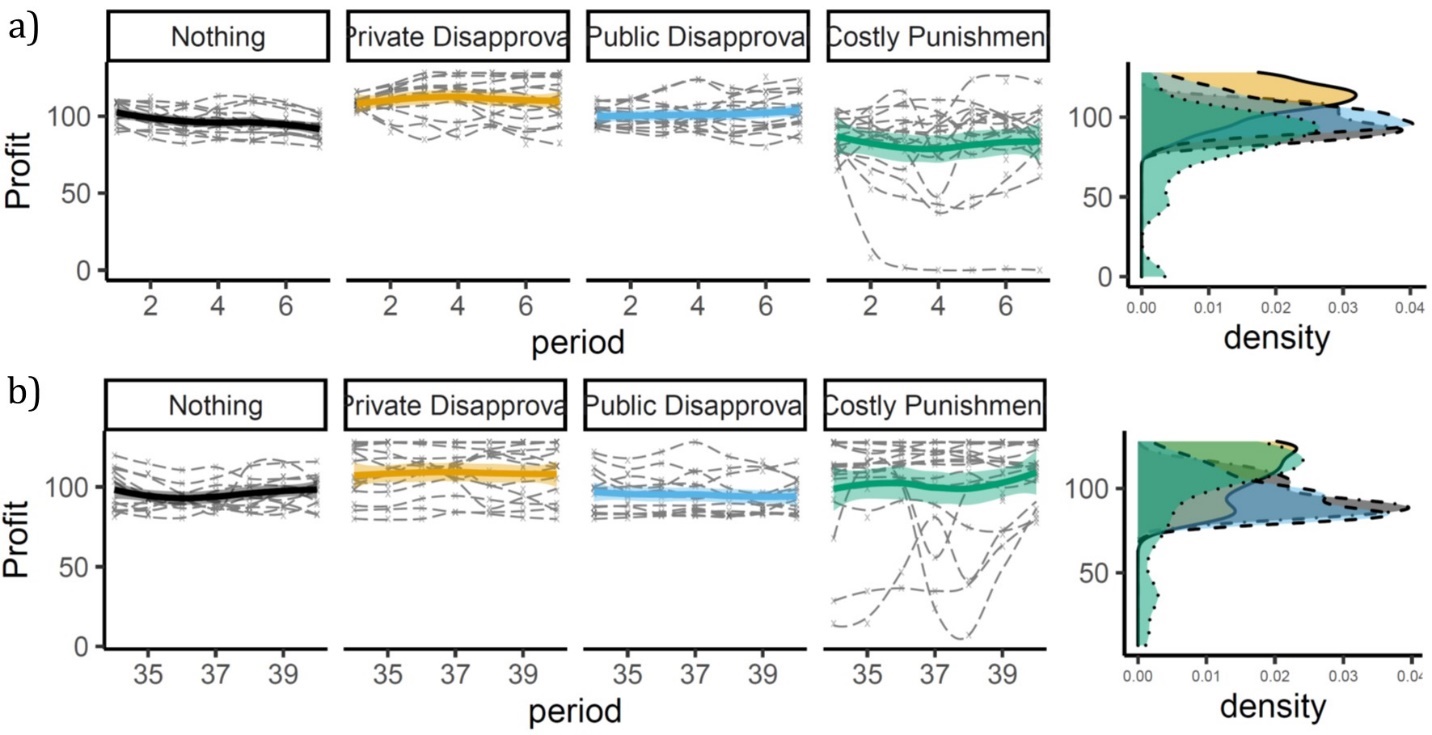


Figure S4.2: Detailed comparison of profits with 95% confidence intervals in a) early periods and b) late periods. In the scatterplots, each ‘x’ is a group’s total contribution; colored solid lines and shaded 95% confidence intervals summarize period mean trends within each condition using LOESS smoothing; grey dashed lines summarize group mean trends with LOESS. Density plots summarize these data, collapsing across period ranges within condition.

## S4.3 Post Hoc Comparisons of Profits in All Conditions

The main text focuses primarily on our planned comparisons of Costly Punishment vs. Public Disapproval, Costly Punishment vs. Nothing, and Public Disapproval vs. Nothing, as these were the most important comparisons for our hypotheses. Here we present post hoc comparisons of earnings per round in all conditions using Tukey HSD, Scheffe, and Sidak tests from our Repeated Measures GLM in SPSS with the four between-subjects conditions. The results are comparable for all three different types of post hoc tests.

### S4.3.1 Post Hoc Tests of Earnings Per Round Across all 40 rounds

*Table S4.3.1 Post hoc tests of profits in all 40 rounds*

| **Multiple Comparisons** | | | | | | | |
| --- | --- | --- | --- | --- | --- | --- | --- |
| Measure: MEASURE_1 | | | | | | | |
|  | (I) condition | (J) condition | Mean Difference (I-J) | Std. Error | Sig. | 95% Confidence Interval | |
|  |  |  |  |  |  | Lower Bound | Upper Bound |
| Tukey HSD | CostPun | NoDis | -3.1216 | 5.75996 | .948 | -18.4091 | 12.1659 |
|  |  | PrivDis | -13.9446 | 5.51985 | .068 | -28.5948 | .7056 |
|  |  | PubDis | -4.2267 | 5.51985 | .869 | -18.8770 | 10.4235 |
|  | NoDis | CostPun | 3.1216 | 5.75996 | .948 | -12.1659 | 18.4091 |
|  |  | PrivDis | -10.8230 | 5.93366 | .274 | -26.5716 | 4.9255 |
|  |  | PubDis | -1.1052 | 5.93366 | .998 | -16.8537 | 14.6433 |
|  | PrivDis | CostPun | 13.9446 | 5.51985 | .068 | -.7056 | 28.5948 |
|  |  | NoDis | 10.8230 | 5.93366 | .274 | -4.9255 | 26.5716 |
|  |  | PubDis | 9.7179 | 5.70088 | .332 | -5.4128 | 24.8485 |
|  | PubDis | CostPun | 4.2267 | 5.51985 | .869 | -10.4235 | 18.8770 |
|  |  | NoDis | 1.1052 | 5.93366 | .998 | -14.6433 | 16.8537 |
|  |  | PrivDis | -9.7179 | 5.70088 | .332 | -24.8485 | 5.4128 |
| Scheffe | CostPun | NoDis | -3.1216 | 5.75996 | .961 | -19.7635 | 13.5204 |
|  |  | PrivDis | -13.9446 | 5.51985 | .108 | -29.8929 | 2.0037 |
|  |  | PubDis | -4.2267 | 5.51985 | .899 | -20.1750 | 11.7215 |
|  | NoDis | CostPun | 3.1216 | 5.75996 | .961 | -13.5204 | 19.7635 |
|  |  | PrivDis | -10.8230 | 5.93366 | .354 | -27.9669 | 6.3208 |
|  |  | PubDis | -1.1052 | 5.93366 | .998 | -18.2490 | 16.0387 |
|  | PrivDis | CostPun | 13.9446 | 5.51985 | .108 | -2.0037 | 29.8929 |
|  |  | NoDis | 10.8230 | 5.93366 | .354 | -6.3208 | 27.9669 |
|  |  | PubDis | 9.7179 | 5.70088 | .415 | -6.7534 | 26.1892 |
|  | PubDis | CostPun | 4.2267 | 5.51985 | .899 | -11.7215 | 20.1750 |
|  |  | NoDis | 1.1052 | 5.93366 | .998 | -16.0387 | 18.2490 |
|  |  | PrivDis | -9.7179 | 5.70088 | .415 | -26.1892 | 6.7534 |
| Sidak | CostPun | NoDis | -3.1216 | 5.75996 | .995 | -18.8746 | 12.6315 |
|  |  | PrivDis | -13.9446 | 5.51985 | .085 | -29.0410 | 1.1518 |
|  |  | PubDis | -4.2267 | 5.51985 | .971 | -19.3232 | 10.8697 |
|  | NoDis | CostPun | 3.1216 | 5.75996 | .995 | -12.6315 | 18.8746 |
|  |  | PrivDis | -10.8230 | 5.93366 | .369 | -27.0512 | 5.4051 |
|  |  | PubDis | -1.1052 | 5.93366 | 1.000 | -17.3333 | 15.1230 |
|  | PrivDis | CostPun | 13.9446 | 5.51985 | .085 | -1.1518 | 29.0410 |
|  |  | NoDis | 10.8230 | 5.93366 | .369 | -5.4051 | 27.0512 |
|  |  | PubDis | 9.7179 | 5.70088 | .448 | -5.8737 | 25.3094 |
|  | PubDis | CostPun | 4.2267 | 5.51985 | .971 | -10.8697 | 19.3232 |
|  |  | NoDis | 1.1052 | 5.93366 | 1.000 | -15.1230 | 17.3333 |
|  |  | PrivDis | -9.7179 | 5.70088 | .448 | -25.3094 | 5.8737 |
| Based on observed means.  The error term is Mean Square(Error) = 227.500. | | | | | | | |

CostPun = Costly Punishment

NoDis = Nothing (i.e., No Disapproval or Punishment)

PrivDis = Private Disapproval

PubDis = Public Disapproval

### S4.3.2 Post Hoc Tests of Earnings Per Round in Early Rounds (i.e., Rounds 1-7)

*Table S4.3.2 Post hoc tests of profits in the early rounds (i.e., rounds 1-7)*

| **Multiple Comparisons** | | | | | | | |
| --- | --- | --- | --- | --- | --- | --- | --- |
| Measure: MEASURE_1 | | | | | | | |
|  | (I) condition | (J) condition | Mean Difference (I-J) | Std. Error | Sig. | 95% Confidence Interval | |
|  |  |  |  |  |  | Lower Bound | Upper Bound |
| Tukey HSD | CostPun | NoDis | -14.2498 | 5.55268 | .062 | -28.9872 | .4875 |
|  |  | PrivDis | -28.3427^*^ | 5.32121 | <.001 | -42.4657 | -14.2197 |
|  |  | PubDis | -18.9570^*^ | 5.32121 | .004 | -33.0800 | -4.8339 |
|  | NoDis | CostPun | 14.2498 | 5.55268 | .062 | -.4875 | 28.9872 |
|  |  | PrivDis | -14.0929 | 5.72013 | .078 | -29.2747 | 1.0889 |
|  |  | PubDis | -4.7071 | 5.72013 | .843 | -19.8889 | 10.4747 |
|  | PrivDis | CostPun | 28.3427^*^ | 5.32121 | <.001 | 14.2197 | 42.4657 |
|  |  | NoDis | 14.0929 | 5.72013 | .078 | -1.0889 | 29.2747 |
|  |  | PubDis | 9.3857 | 5.49573 | .330 | -5.2005 | 23.9719 |
|  | PubDis | CostPun | 18.9570^*^ | 5.32121 | .004 | 4.8339 | 33.0800 |
|  |  | NoDis | 4.7071 | 5.72013 | .843 | -10.4747 | 19.8889 |
|  |  | PrivDis | -9.3857 | 5.49573 | .330 | -23.9719 | 5.2005 |
| Scheffe | CostPun | NoDis | -14.2498 | 5.55268 | .100 | -30.2929 | 1.7933 |
|  |  | PrivDis | -28.3427^*^ | 5.32121 | <.001 | -43.7170 | -12.9683 |
|  |  | PubDis | -18.9570^*^ | 5.32121 | .009 | -34.3313 | -3.5826 |
|  | NoDis | CostPun | 14.2498 | 5.55268 | .100 | -1.7933 | 30.2929 |
|  |  | PrivDis | -14.0929 | 5.72013 | .122 | -30.6198 | 2.4341 |
|  |  | PubDis | -4.7071 | 5.72013 | .878 | -21.2341 | 11.8198 |
|  | PrivDis | CostPun | 28.3427^*^ | 5.32121 | <.001 | 12.9683 | 43.7170 |
|  |  | NoDis | 14.0929 | 5.72013 | .122 | -2.4341 | 30.6198 |
|  |  | PubDis | 9.3857 | 5.49573 | .413 | -6.4928 | 25.2643 |
|  | PubDis | CostPun | 18.9570^*^ | 5.32121 | .009 | 3.5826 | 34.3313 |
|  |  | NoDis | 4.7071 | 5.72013 | .878 | -11.8198 | 21.2341 |
|  |  | PrivDis | -9.3857 | 5.49573 | .413 | -25.2643 | 6.4928 |
| Sidak | CostPun | NoDis | -14.2498 | 5.55268 | .077 | -29.4360 | .9364 |
|  |  | PrivDis | -28.3427^*^ | 5.32121 | <.001 | -42.8958 | -13.7895 |
|  |  | PubDis | -18.9570^*^ | 5.32121 | .005 | -33.5101 | -4.4038 |
|  | NoDis | CostPun | 14.2498 | 5.55268 | .077 | -.9364 | 29.4360 |
|  |  | PrivDis | -14.0929 | 5.72013 | .098 | -29.7370 | 1.5513 |
|  |  | PubDis | -4.7071 | 5.72013 | .960 | -20.3513 | 10.9370 |
|  | PrivDis | CostPun | 28.3427^*^ | 5.32121 | <.001 | 13.7895 | 42.8958 |
|  |  | NoDis | 14.0929 | 5.72013 | .098 | -1.5513 | 29.7370 |
|  |  | PubDis | 9.3857 | 5.49573 | .446 | -5.6447 | 24.4161 |
|  | PubDis | CostPun | 18.9570^*^ | 5.32121 | .005 | 4.4038 | 33.5101 |
|  |  | NoDis | 4.7071 | 5.72013 | .960 | -10.9370 | 20.3513 |
|  |  | PrivDis | -9.3857 | 5.49573 | .446 | -24.4161 | 5.6447 |
| Based on observed means.  The error term is Mean Square(Error) = 211.421. | | | | | | | |
| *. The mean difference is significant at the .05 level. | | | | | | | |

CostPun = Costly Punishment

NoDis = Nothing (i.e., No Disapproval or Punishment)

PrivDis = Private Disapproval

PubDis = Public Disapproval

### S4.3.3 Post Hoc Tests of Earnings Per Round in Late Rounds (i.e., Rounds 34-40)

*Table S4.3.3 Post hoc tests of profits in the late rounds (i.e., rounds 34-40)*

| **Multiple Comparisons** | | | | | | | |
| --- | --- | --- | --- | --- | --- | --- | --- |
| Measure: MEASURE_1 | | | | | | | |
|  | (I) condition | (J) condition | Mean Difference (I-J) | Std. Error | Sig. | 95% Confidence Interval | |
|  |  |  |  |  |  | Lower Bound | Upper Bound |
| Tukey HSD | CostPun | NoDis | 6.0543 | 6.71486 | .804 | -11.7676 | 23.8762 |
|  |  | PrivDis | -6.4263 | 6.43495 | .751 | -23.5053 | 10.6527 |
|  |  | PubDis | 6.9818 | 6.43495 | .700 | -10.0972 | 24.0608 |
|  | NoDis | CostPun | -6.0543 | 6.71486 | .804 | -23.8762 | 11.7676 |
|  |  | PrivDis | -12.4806 | 6.91737 | .283 | -30.8400 | 5.8788 |
|  |  | PubDis | .9276 | 6.91737 | .999 | -17.4318 | 19.2869 |
|  | PrivDis | CostPun | 6.4263 | 6.43495 | .751 | -10.6527 | 23.5053 |
|  |  | NoDis | 12.4806 | 6.91737 | .283 | -5.8788 | 30.8400 |
|  |  | PubDis | 13.4082 | 6.64599 | .195 | -4.2310 | 31.0473 |
|  | PubDis | CostPun | -6.9818 | 6.43495 | .700 | -24.0608 | 10.0972 |
|  |  | NoDis | -.9276 | 6.91737 | .999 | -19.2869 | 17.4318 |
|  |  | PrivDis | -13.4082 | 6.64599 | .195 | -31.0473 | 4.2310 |
| Scheffe | CostPun | NoDis | 6.0543 | 6.71486 | .846 | -13.3467 | 25.4552 |
|  |  | PrivDis | -6.4263 | 6.43495 | .802 | -25.0186 | 12.1659 |
|  |  | PubDis | 6.9818 | 6.43495 | .759 | -11.6104 | 25.5741 |
|  | NoDis | CostPun | -6.0543 | 6.71486 | .846 | -25.4552 | 13.3467 |
|  |  | PrivDis | -12.4806 | 6.91737 | .364 | -32.4667 | 7.5054 |
|  |  | PubDis | .9276 | 6.91737 | .999 | -19.0585 | 20.9136 |
|  | PrivDis | CostPun | 6.4263 | 6.43495 | .802 | -12.1659 | 25.0186 |
|  |  | NoDis | 12.4806 | 6.91737 | .364 | -7.5054 | 32.4667 |
|  |  | PubDis | 13.4082 | 6.64599 | .266 | -5.7938 | 32.6101 |
|  | PubDis | CostPun | -6.9818 | 6.43495 | .759 | -25.5741 | 11.6104 |
|  |  | NoDis | -.9276 | 6.91737 | .999 | -20.9136 | 19.0585 |
|  |  | PrivDis | -13.4082 | 6.64599 | .266 | -32.6101 | 5.7938 |
| Sidak | CostPun | NoDis | 6.0543 | 6.71486 | .938 | -12.3104 | 24.4190 |
|  |  | PrivDis | -6.4263 | 6.43495 | .903 | -24.0255 | 11.1728 |
|  |  | PubDis | 6.9818 | 6.43495 | .864 | -10.6173 | 24.5810 |
|  | NoDis | CostPun | -6.0543 | 6.71486 | .938 | -24.4190 | 12.3104 |
|  |  | PrivDis | -12.4806 | 6.91737 | .382 | -31.3991 | 6.4379 |
|  |  | PubDis | .9276 | 6.91737 | 1.000 | -17.9910 | 19.8461 |
|  | PrivDis | CostPun | 6.4263 | 6.43495 | .903 | -11.1728 | 24.0255 |
|  |  | NoDis | 12.4806 | 6.91737 | .382 | -6.4379 | 31.3991 |
|  |  | PubDis | 13.4082 | 6.64599 | .259 | -4.7682 | 31.5845 |
|  | PubDis | CostPun | -6.9818 | 6.43495 | .864 | -24.5810 | 10.6173 |
|  |  | NoDis | -.9276 | 6.91737 | 1.000 | -19.8461 | 17.9910 |
|  |  | PrivDis | -13.4082 | 6.64599 | .259 | -31.5845 | 4.7682 |
| Based on observed means.  The error term is Mean Square(Error) = 309.184. | | | | | | | |

CostPun = Costly Punishment

NoDis = Nothing (i.e., No Disapproval or Punishment)

PrivDis = Private Disapproval

PubDis = Public Disapproval
